# Hypertension treatment cascade in India: results from National Noncommunicable Disease Monitoring Survey

**DOI:** 10.1038/s41371-022-00692-y

**Published:** 2022-05-05

**Authors:** Ritvik Amarchand, Vaitheeswaran Kulothungan, Anand Krishnan, Prashant Mathur

**Affiliations:** 1 Centre for Community Medicine, All India Institute of Medical Sciences, New Delhi, India; 2 Indian Council Medical Research (ICMR)—National Centre for Disease Informatics and Research (NCDIR), Bengaluru, Karnataka India

**Keywords:** Risk factors, Hypertension

## Abstract

Hypertension is a major risk factor for ischemic heart disease and stroke. We estimated prevalence, awareness, treatment, and control of hypertension along with its determinants in India. We used data from the National NCD Monitoring Survey-(NNMS-2017-2018) which studied one adult (18–69 years) from a representative sample of households across India and collected information on socio-demographic variables, risk factors for NCDs and treatment practices. Blood pressure was recorded digitally and hypertension was defined as systolic blood pressure (SBP) ≥ 140 mmHg or diastolic blood pressure (DBP) ≥ 90 mmHg or currently on medications. Awareness was defined as being previously diagnosed with hypertension by a health professional; on treatment as taking a dose of medication once in the last 14 days and; control as SBP < 140 mmHg and DBP < 90 mmHg. Multivariate Logistic regression was performed to estimate determinants. Out of 10,593 adults with a blood pressure measurement (99.4%), 3017 (28.5%; 95% CI: 27.0–30.1) were found to have hypertension. Of these hypertensives, 840 (27.9%; 95% CI: 25.5–30.3) were aware, 438 (14.5%; 95% CI: 12.7–16.5) were under treatment and, 379 (12.6%; 95% CI: 11.0–14.3) were controlled. Significant determinants of awareness were being in the age group 50–69 years (aOR 2.45 95% CI: 1.63–3.69), women (1.63; 95% CI: 1.20–2.22) and from higher wealth quintiles. Those in the age group 50–69 (aOR 4.80; 95% CI: 1.74–13.27) were more likely to be under treatment. Hypertension control was poorer among urban participants (aOR 0.55; 95% CI: 0.33–0.90). Significant regional differences were noted, though without any clear trend. One-fifth of the patients were being managed at public facilities. The poor population-level hypertension control needs strengthening of hypertension services in the Universal Health Coverage package.

## Introduction

Hypertension is one of the most important risk factors for cardiovascular diseases (CVD), particularly ischemic heart disease and stroke [1, 2]. Currently, it is estimated that 28.1% of all deaths in India were due to CVD and, high systolic blood pressure (SBP) was the single largest contributor (8.5%) to disability-adjusted life years DALYs [3]. The World Health Organization target of a 25% relative reduction in the prevalence of high blood pressure (BP) among persons aged 18 years and older by 2025 can only be achieved by a combination of strategies that shift the population distribution of BP to the left and achieve good control of BP among those with hypertension [4, 5]. A good understanding of the cascade of hypertension of “awareness,” “treatment,” and “control” is useful to plan hypertension control strategy in a population as these have different implications [6–8].

Based on early experience in the United States, a rule of halves was postulated to hold with 50% of those with hypertension being aware of it, 50% of them being treated and 50% of those treated being controlled [9]. However, its universal applicability has been questioned [10]. A nationwide survey in India among 18–49 years in 2015–2016 showed that in those with high BP, 44.7% were aware of their diagnosis, 13.3% were being treated, and 7.9% had achieved control [11]. In a secondary data analysis of National Family Health Survey (NFHS 2015-16) data. the prevalence of hypertension among men aged 15–54 years was 16%. Of these hypertensive individuals, 63.2% had their BP measured earlier, 21.5% were aware of the diagnosis, 12.6% were treated and only 6.1% had controlled BP [12]. The estimated prevalence of hypertension for the Indian population aged 45 years and older, studied as a part of a longitudinal study on ageing, was 45.9%, with 55.7% of hypertension being already diagnosed, 38.9% were on antihypertensive medication and 31.7% had their BP under control [13]. A recent study in which these parameters were estimated 20 years apart in the National Capital Region of Delhi showed that there was little change in these parameters in an urban area while, the awareness, treatment and control had marginally improved in the rural population, though control of hypertension was still poorer than in urban area [14]. While these surveys provide useful information, they do not cover the age groups (18-69 years) needed for global and national monitoring efforts.

India recently strengthened its resolve to address hypertension at the population level by launching the population-based screening for hypertension (along with diabetes and the three common cancers) and strengthening primary and secondary health facilities [15, 16]. The National NCD Monitoring Survey (NNMS) in 2017–2018 was conducted to monitor the progress of the NCD monitoring indicators [17]. This paper presents the results related to hypertension cascade—prevalence, awareness, treatment and control.

## Materials and methods

The NNMS was conducted in 2017–2018 in covering a total of 300 rural (village) and 300 urban (ward) clusters, selected by multi-stage stratified random sample, and 20 households were selected in each of the clusters using systematic random sampling (Supplementary Fig. 1a). In each selected household, one adult member (18–69 years) was selected for the study. The details of the survey process including the selection of households and individuals and study tools have been published earlier [17]. The survey was implemented by ten regional partner institutions and followed standard operating procedures and training protocols. Automatic BP machines (OMRON HEM–7120, Omron Corporation, Kyoto, Japan) were used with regular calibration being done at the study sites.

Information collected were household-level data on socioeconomic status, fuel and cooking oil use, individual-level data on demographic details, socio-behavioral risk factors for NCDs; diagnosis and treatment-seeking for hypertension, diabetes and CVD. Anthropometric (height, weight, and waist circumference), BP and blood glucose measurements were also carried out on the participant selected for the survey. BP was recorded in the left arm in a seated position after resting the person for 5–10 min. Three readings were taken at least 5 min apart.

The definitions used for BP followed standard recommendations of the World Hypertension League Expert Committee [8]. The proportion of participants who reported their BP being checked at least once by a physician or a health worker in their lifetime were labeled as ever measured. High BP was defined as systolic blood pressure (SBP) ≥ 140 mmHg or diastolic blood pressure (DBP) ≥ 90 mmHg (based on the mean of the 2nd and 3rd measurements of BP based on the minimum percentage regression to mean (SBP: 5.8% and DBP: 9.9%) compared to other two measurement combination) or the participants who reported being currently on medications for raised BP or who reported having been diagnosed with hypertension by a health professional. The participants with high BP who reported having been diagnosed with hypertension by a health professional or who report taking medication for high BP were classified as being aware. The proportion of participants with hypertension who reported taking medication for high BP on any of the last 2 weeks before the survey day were considered to be on treatment. Hypertension control was defined as having an SBP < 140 mmHg and DBP < 90 mmHg. Presence of concomitant behavioral risk factors [current smoked or smokeless tobacco use, ever intake of alcohol and hazardous drinking (more than 6 standard drinks in a single drinking occasion in last 30 days), physical inactivity (less than 600 MET-minutes in a week), dietary salt restriction practices] co-morbidities (diabetes, hypercholesterolemia or, CVD), increased body mass index and increased waist circumference (≥90 cm in males and ≥80 cm in females), treatment-seeking practices, adherence to medication (number of days medication taken in last 14 days categorized into three groups 1–5 days, 6–10 days and 11 or more days) and source of drugs for hypertension (government/ others) were all explored as determinants of hypertension control and for estimation of crude and adjusted odds ratio and 95% CI.

The survey was done using an offline android-based application, Open Data Kit. The finalized forms, after review by the team leader for completion, were uploaded at the end of the day or the survey cluster. Data was cleaned in SPSS Version 22.0 and weighted for adjusting sample, population proportions and response rates to provide nationally representative prevalence estimates at the population level. The information on household possession of select assets was used to calculate the wealth index of all surveyed households and divided into quintiles. Final weighted data was analyzed in STATA 14.1 by complex survey analysis and the population estimates of hypertension treatment cascade are presented as proportion with 95% confidence intervals (CIs). These proportions and 95% CI were derived for different subgroups of age, sex, years of education, profession, wealth index quintiles, region, place of residence (urban or rural). Participants were categorized based on the regions of the country in which their state was present—Central (Uttar Pradesh, Chhattisgarh, Madhya Pradesh); East (Bihar, West Bengal, Jharkhand, Odisha); North (Jammu and Kashmir, Himachal Pradesh, Punjab, Chandigarh, Uttarakhand, Haryana, Delhi, Rajasthan); South (Andhra Pradesh, Karnataka, Kerala, Tamil Nadu); West (Gujarat, Maharashtra); and North-East (Sikkim, Nagaland, Manipur, Mizoram, Assam) [18].

Multivariate logistic regression was performed to estimate adjusted odds ratios with their 95% CI with every measurement, prevalence, awareness, treatment and control of hypertension as dependent variables and the above variables as an independent. We also compared the management practices reported by the subjects with hypertension (source of treatment and medicines, receipt of advice on lifestyle management and level of adherence to medications) by place of residence.

## Results

**Fig. 1:**
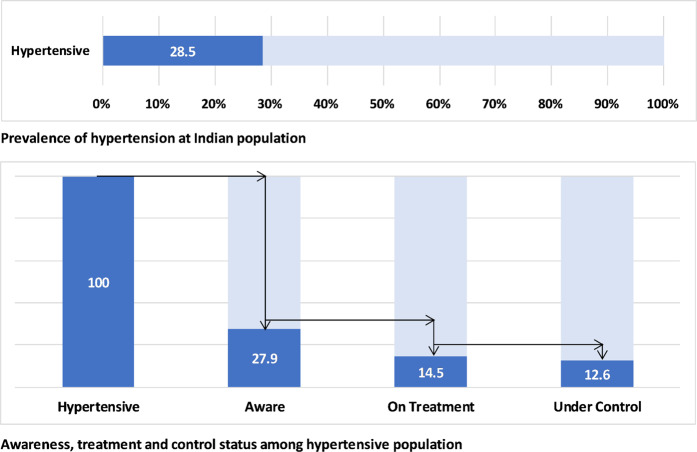
Hypertension control cascade: gap in prevalence, awareness, treatment and control of hypertension. Panel 1: Prevalence of hypertension at Indian population. Panel 2: Awareness, treatment and control status among hypertensive population.

A total of 10,659 individuals provided complete information in the NNMS survey (96.3%), of which 10,593 had their BP measured (99.4%). Total males were 5490 (99.1%), 5103 were females (99.7%), urban adults were 3538 (99.1%) and 7055 rural adults (99.5%) (Supplementary Fig. 1b). Of the total adults, 3017 (28.5%; 95% CI: 27.0–30.1) were found to have high BP based on their BP measurement values and for reported history of being treated for hypertension. Of them, 840 (27.9%; 95% CI: 25.5–30.3) were aware of their hypertension status, 438 (14.5%; 95% CI: 12.7–16.5) were under treatment for hypertension and, 379 (12.6%; 95% CI: 11.0–14.3) had their BP under control. (Fig. 1).

Among the surveyed adults, 47.6% (95% CI: 45.2–50.0) reported having their BP measured ever in their lifetime (Table 1). This was significantly more among women as compared to men (aOR 1.92; 95% CI: 1.59–2.32), among those aged >30 years as compared to those between 18–30 years. The BP measurement showed a clear socioeconomic patterning with those educated beyond sixth class or those not working or professionals or people in higher wealth index being significantly more likely to have their BP measured. Adults living in southern India (aOR 2.41; 95% CI: 1.88–3.09) were more likely to have their BP measured, while the rural-urban differences ceased to be significant after adjustment.

**Table 1:** Measurement of blood pressure and prevalence of high blood pressure and their determinants in Indian population.

Subgroups	Ever measured blood pressure				High blood pressure		
	*n*	Prevalence (%)	UOR	AOR	Prevalence (%)	UOR	AOR
		(95% CI)	(95% CI)	(95% CI)	(95% CI)	(95% CI)	(95% CI)
Total	10,593	47.6			28.5		
		(45.2–50.0)			(27.0–30.1)		
Age groups (in years)							
18–29	3125	36.2	1	1	13.2	1	1
		(33.4–39.1)			(11.5–15.0)		
30–49	5121	48.2	1.64	1.73	28	2.55	2.38
		(45.5–50.9)	(1.45–1.86)	(1.50–2.00)	(25.9–30.1)	(2.15–3.04)	(1.99–2.85)
50–69	2347	61.3	2.8	3.11	50	6.58	6.02
		(57.7–64.9)	(2.38–3.30)	(2.60–3.72)	(47.1–52.9)	(5.46–7.94)	(4.92–7.36)
Sex							
Male	5490	40.9	1	1	29.9	1	1
		(38.0–43.8)			(27.9–32.0)		
Female	5103	54.8	1.75	**1.92**	27	0.87	**0.82**
		(51.9–57.6)	(1.54–2.01)	**(1.59–2.32)**	(25.2–28.8)	(0.77–0.97)	**(0.71–0.95)**
Education^a^							
Primary	4797	41.9	1	1	30.9	1	1
		(38.8–45.0)			(28.8–33.2)		
Secondary	3350	50.6	1.42	1.47	26.3	0.8	0.89
		(47.7–53.6)	(1.24–1.63)	(1.27–1.70)	(23.9–29.0)	(0.69–0.92)	(0.76–1.04)
Higher secondary and above	2436	54.6	1.67	1.31	26.5	0.81	0.88
		(51.2–57.9)	(1.41–1.98)	(1.06–1.62)	(24.2–28.9)	(0.69–0.94)	(0.72–1.07)
Occupation							
Skilled/unskilled laborers	3606	36.8	1	1	27	1	1
		(33.6–40.0)			(24.6–29.6)		
Not working^b^	4609	53.4	1.97	1.36	26.9	0.99	1.05
		(50.5–56.2)	(1.70–2.28)	(1.12–1.66)	(25.0–28.8)	(0.86–1.14)	(0.87–1.25)
Others^c^	2367	52.9	1.93	1.32	33.9	1.39	1.16
		(48.9–56.8)	(1.61–2.31)	(1.08–1.62)	(31.2–36.8)	(1.17–1.65)	(0.97–1.40)
Wealth index quintiles							
Q1	2815	29.9	1	1	25.7	1	1
		(26.7–33.3)			(23.1–28.5)		
Q2	2421	43.1	1.78	1.69	26	1.01	1.06
		(39.7–46.5)	(1.51–2.09)	(1.44–1.98)	(23.3–28.8)	(0.86–1.20)	(0.89–1.27)
Q3	2062	49.2	2.27	1.89	25.7	1	0.99
		(45.9–52.5)	(1.90–2.72)	(1.56–2.27)	(22.7–28.9)	(0.81–1.23)	(0.79–1.26)
Q4	1759	58.9	3.35	2.65	32.5	1.4	1.25
		(55.3–62.3)	(2.71–4.15)	(2.10–3.34)	(29.5–35.8)	(1.15–1.69)	(0.99–1.57)
Q5	1536	72	6.02	4.69	36.7	1.68	1.35
		(68.2–75.4)	(4.76–7.61)	(3.53–6.23)	(34.1–39.4)	(1.41–2.00)	(1.05–1.72)
Regions of India							
Central	2663	39.7	1	1	23	1	1
		(35.6–44.0)			(20.3–26.0)		
East	2343	36.8	0.88	0.99	27.1	1.24	1.25
		(32.7–41.2)	(0.69–1.14)	(0.78–1.26)	(23.3–31.2)	(0.96–1.60)	(0.96–1.63)
North	1391	51.1	1.58	1.08	33.7	1.7	**1.52**
		(44.1–58.0)	(1.14–2.20)	(0.83–1.40)	(29.7–37.9)	(1.33–2.17)	**(1.20–1.93)**
South	2537	65.3	2.85	**2.41**	33.6	1.7	**1.36**
		(61.3–69.1)	(2.22–3.66)	**(1.88–3.09)**	(30.8–36.6)	(1.38–2.09)	**(1.11–1.67)**
West	1176	43.6	1.17	1	26	1.17	1.07
		(38.4–48.9)	(0.89–1.55)	(0.75–1.32)	(22.5–29.7)	(0.92–1.50)	(0.84–1.35)
North-East	483	49.4	1.48	1.16	29.4	1.39	1.19
		(40.6–58.3)	(0.99–2.21)	(0.89–1.52)	(24.9–34.2)	(1.05–1.83)	(0.90–1.57)
Place of residence							
Rural	7055	41.4	1	1	25.7	1	1
		(38.5–44.3)			(23.8–27.8)		
Urban	3538	59.9	2.12	1.05	34	1.49	1.29
		(56.5–63.2)	(1.76–2.54)	(0.87–1.26)	(32.0–36.1)	(1.30–1.71)	(1.10–1.51)

Bold values indicate statistical significance *p* < 0.05.

^a^Primary: illiterate and <6th standard; Secondary: 6th to 10th standard; Higher secondary and above: 11th standard and above.

^b^Homemaker/retired/unemployed.

^c^Professionals/managers/executives/self-employed.

Women were less likely to have high BP (aOR 0.82; 95% CI: 0.71–0.95) as were people in the lower age brackets (Table 1). Education and occupation were not found to be significant determinants of high BP. Those belonging to the highest wealth quintiles (Q5) had a significantly higher prevalence of high BP (aOR:1.35, 95% CI: 1.05–1.72). People in the northern (aOR 1.52; 95% CI: 1.20–1.93) and southern (aOR 1.35; 95% CI: 1.10–1.66) India were more likely to have high BP as compared to those in the central region. Urban residents were also found to have significantly high BP (aOR 1.29; 95% CI: 1.10–1.51) as compared to rural ones.

Table 2 presents the data on the awareness, treatment and control rates among those with hypertension and their determinants. Awareness of their high BP status was higher among those aged 50–69 years (aOR 2.45; 95% CI: 1.63–3.69); among women (aOR 1.63; 95% CI: 1.20–2.22), among those not working and professionals as compared to laborers, and those in the higher wealth index groups. Those living in the western region of India were less likely to be aware of their hypertension status (aOR 0.61; 95% CI: 0.40–0.94) whereas those in the northeast were more likely to be aware of their BP status (aOR 1.71; 95% CI: 1.02–2.85). There was no significant rural-urban differences on this parameter.

**Table 2:** Prevalence (%) and determinants (aOR with 95% CI) of awareness, treatment, and control among those with hypertension.

Subgroups	Awareness				Treatment			Control		
	*n*	Prevalence (%)	UOR	AOR	Prevalence (%)	UOR	AOR	Prevalence (%)	UOR	AOR
		(95% CI)	(95% CI)	(95% CI)	(95% CI)	(95% CI)	(95% CI)	(95% CI)	(95% CI)	(95% CI)
Total	3017	27.9			14.5			12.6		
		(25.5–30.3)			(12.7–16.5)			(11.0–14.3)		
Age groups (in years)										
18–29	412	19.3	1	1	5.3	1	1	14.1	1	1
		(14.4–25.3)			(2.4–11.2)			(9.8–19.8)		
30–49	1431	22.3	1.21	1.23	9.4	1.9	2.09	10.8	0.59	0.95
		(19.3–25.8)	(0.79–1.83)	(0.80–1.88)	(7.3–12.0)	(0.75–4.85)	(0.77–5.71)	(8.5–13.6)	(0.11–3.18)	(0.21–4.35)
50–69	1174	37.6	2.52	**2.45**	24	4.6	**4.8**	14.2	0.32	0.54
		(33.8–41.5)	(1.73–3.69)	**(1.63–3.69)**	(20.7–27.6)	(1.78–11.89)	**(1.74–13.27)**	(11.9–16.9)	(0.07–1.57)	(0.13–2.32)
Sex										
Male	1642	22.6	1	1	10.9	1	1	10.3	1	1
		(19.8–25.7)			(8.9–13.3)			(8.2–12.8)		
Female	1375	34.1	1.77	**1.63**	18.8	1.33	1.28	15.3	0.64	0.57
		(30.7–37.7)	(1.42–2.20)	**(1.20–2.22)**	(16.2–21.7)	(0.92–1.91)	(0.75–2.19)	(13.0–17.8)	(0.35–1.17)	(0.31–1.06)
Education^a^										
Primary	1484	27.5	1	1	14.4	1	1	13	1	1
		(24.7–30.4)			(12.2–16.8)			(11.0–15.4)		
Secondary	882	25.9	0.92	0.96	14.4	1.15	1.16	10.7	0.88	0.84
		(21.8–30.5)	(0.72–1.19)	(0.71–1.30)	(11.3–18.2)	(0.73–1.80)	(0.70–1.92)	(8.3–13.7)	(0.50–1.56)	(0.43–1.63)
Higher secondary and above	646	31.3	1.21	1.16	15.1	0.85	1.18	14.1	1.17	1.02
		(26.7–36.3)	(0.94–1.54)	(0.81–1.66)	(11.6–19.4)	(0.52–1.38)	(0.58–2.41)	(10.3–19.2)	(0.56–2.45)	(0.42–2.47)
Occupation										
Skilled/unskilled laborers	974	17.1	1	1	7.6	1	1	8.5	1	1
		(14.1–20.5)			(5.6–10.3)			(6.5–11.1)		
Not working^b^	1238	36.1	2.74	**1.59**	20	1.53	1.35	16.3	0.83	1.28
		(32.6–39.8)	(2.09–3.60)	**(1.13–2.23)**	(17.2–23.1)	(0.94–2.50)	(0.72–2.55)	(13.8–19.1)	(0.44–1.58)	(0.65–2.57)
Others^c^	803	28.2	1.91	1.39	14.4	1.29	1.39	11.8	1.04	0.9
		(23.5–33.5)	(1.38–2.64)	(0.98–1.96)	(10.7–19.3)	(0.71–2.36)	(0.67–2.88)	(8.4–16.3)	(0.43–2.50)	(0.37–2.23)
Wealth index quintiles										
Q1	723	17.3	1	1	**6.7**	1	1	9.4	1	1
		(13.9–21.2)			**(4.4–9.9)**			(6.9–12.7)		
Q2	628	26.2	1.7	**1.87**	13.7	1.74	1.67	12.1	0.9	0.82
		(21.8–31.0)	(1.19–2.42)	**(1.27–2.74)**	(10.1–18.3)	(0.92–3.30)	(0.80–3.45)	(8.5–16.9)	(0.32–2.50)	(0.29–2.29)
Q3	530	23.5	1.47	**1.59**	12.7	1.88	1.63	11.2	0.89	0.92
		(19.2–28.4)	(1.02–2.12)	**(1.07–2.35)**	(10.0–16.1)	(0.95–3.74)	(0.76–3.47)	(8.5–14.7)	(0.33–2.44)	(0.32–2.69)
Q4	572	33.4	2.4	**2.54**	18.7	2.03	1.99	14.3	0.83	0.87
		(28.8–38.3)	(1.72–3.36)	**(1.74–3.72)**	(15.1–23.0)	(1.11–3.73)	(0.99–4.04)	(10.6–19.0)	(0.34–2.02)	(0.31–2.47)
Q5	564	41.8	3.45	**3.45**	22.9	1.91	1.79	16.6	0.71	0.87
		(36.4–47.5)	(2.42–4.91)	**(2.24–5.30)**	(18.0–28.6)	(0.98–3.73)	(0.77–4.16)	(12.9–21.2)	(0.27–1.87)	(0.29–2.63)
Regions of India										
Central	613	25.9	1	1	8.5	1	1	11.9	1	1
		(21.1–31.5)			(6.3–11.4)			(8.8–15.7)		
East	634	22.7	0.84	0.92	12.1	2.34	**2.65**	12.7	2.16	1.72
		(18.7–27.4)	(0.58–1.21)	(0.63–1.34)	(8.9–16.3)	(1.32–4.14)	**(1.40–5.01)**	(9.3–17.1)	(0.72–6.48)	(0.59–5.04)
North	469	35.6	1.58	1.19	19	2.36	2.01	17.1	1.45	1.46
		(29.7–41.9)	(1.08–2.31)	(0.82–1.73)	(14.1–25.2)	(1.30–4.27)	(0.99–4.07)	(13.0–22.1)	(0.59–3.59)	(0.60–3.57)
South	854	30.6	1.26	1.16	19.7	3.71	**3.01**	11.8	0.84	1.09
		(25.7–35.9)	(0.88–1.80)	(0.80–1.68)	(15.9–24.1)	(2.14–6.42)	**(1.69–5.36)**	(9.2–15.0)	(0.38–1.86)	(0.48–2.48)
West	305	18.4	0.64	**0.61**	11.1	3.1	**3.14**	7.8	0.98	1.02
		(14.0–23.8)	(0.42–0.99)	**(0.40–0.94)**	(7.8–15.5)	(1.48–6.49)	**(1.54–6.40)**	(5.1–11.8)	(0.39–2.43)	(0.38–2.72)
North-East	142	37.6	1.72	**1.71**	12.7	1.04	0.9	15	0.73	0.91
		(26.6–50.2)	(0.97–3.08)	**(1.02–2.85)**	(6.8–22.4)	(0.52–2.08)	(0.42–1.94)	(7.5–27.6)	(0.16–3.39)	(0.21–3.93)
Place of residence										
Rural	1813	26	1	1	12.7	1	1	13.3	1	1
		(23.2–29.1)			(10.3–15.5)			(11.2–15.7)		
Urban	1204	30.6	1.25	0.83	17.3	1.36	1.15	11.4	0.48	**0.55**
		(26.6–34.9)	(0.98–1.60)	(0.63–1.09)	(14.7–20.2)	(0.90–2.07)	(0.73–1.82)	(9.2–14.1)	(0.28–0.81)	**(0.33–0.90)**

Bold values indicate statistical significance *p* < 0.05.

^a^Primary: illiterate and <6th standard; Secondary: 6th to 10th standard; Higher secondary and above: 11th standard and above.

^b^Homemaker/retired/unemployed.

^c^Professionals/managers/executives/self-employed.

There were no significant differences in the proportion of hypertensives on treatment by sex, education, occupation, wealth index, or place of residence. People aged more than 50 years (aOR 4.80; 95% CI: 1.74–13.27) or living in east (aOR 2.65; 95% CI: 1.40–5.01) South (aOR 3.01; 95% CI: 1.69–5.36) or West (aOR 3.14; 95% CI: 1.54–6.40) India had significantly higher treatment rates. People living in urban areas were less likely to have their high BP controlled as compared to the rural areas (aOR 0.55; 95% CI: 0.33–0.90).

Only one-fifth of the subjects with hypertension were being managed at (21.9%; 95% CI: 17.1–27.7) or getting their medicines (18.1%; 95% CI: 13.6–23.8) from a public health facility with no significant rural-urban differences (Table 3). There was very little utilization of the AYUSH (Ayurveda, Yoga, Unani, Siddha, and Homeopathy) system alone in the treatment of hypertension. Good adherence was measured by the reported intake of pills taken in last 2 weeks on at least 80% days (≥11 days’ intake of medicine in last 2 weeks) based on the definition given by Haynes et al. [15]. Good adherence was (70.6%; 95% CI: 63.6–76.7) being significantly higher in urban (83.2%) as compared to rural areas (59.2%). Advice by health care providers regarding behavioral modification was uniformly poor in urban and rural areas. Dietary advice (49.7%) and reducing salt intake (41.6%) were the advice most often given and quitting alcohol (9%) was the least likely advice. Tobacco cessation (13.3%) and increasing physical activity (32.5) were other advice reported.

**Table 3:** Current treatment practices of hypertension in the study subjects.

	Rural		Urban		Combined	
	*n*	% (95% CI)	*n*	% (95% CI)	*n*	% (95% CI)
Source of management						
Govt. health facility	51	22.2	45	21.7	96	21.9
		(15.0–31.6)		(16.0–28.7)		(17.1–27.7)
PVT/NGO health facility	158	68.8	144	69.3	302	69.1
		(58.4–77.6)		(61.4–76.3)		(62.6–74.9)
No	21	9	19	9	40	9
		(4.3–17.8)		(5.4–14.5)		(5.7–13.8)
Source of medicines^a^						
Govt. facility only	42	18.3	38	17.9	80	18.1
		(11.5–28.0)		(12.9–24.3)		(13.6–23.8)
Chemist/Private/NGO Dispensary	180	78.3	164	79	344	78.7
		(69.1–85.3)		(72.7–84.2)		(73.2–83.3)
Both	5	2	2	1.2	7	1.6
		(0.6–6.6)		(0.5–2.8)		(0.7–3.8)
Type of medicines						
Allopathic	193	84.2	166	79.8	359	82.1
		(75.2–90.3)		(72.0–85.9)		(76.3–86.8)
AYUSH medicine	1	0.4	3	1.4	4	0.9
		(0.1–2.7)		(0.4–4.5)		(0.3–2.4)
Both	36	15.4	39	18.7	75	17
		(9.3–24.4)		(13.0–26.4)		(12.5–22.7)
Level of treatment adherence^b^						
≤5 days	39	17.1	9	4.2	48	11
		(10.6–26.3)		(2.0–8.4)		(7.2–16.4)
6–10 days	55	23.7	26	12.6	81	18.4
		(15.3–34.9)		(7.5–20.2)		(13.2–25.2)
≥11 days	136	59.2	173	83.2	309	70.6
		(48.6–68.9)		(75.6–88.8)		(63.6–76.7)
Lifestyle advices^c^						
Quit tobacco	39	17.1	19	9.1	58	13.3
		(11.1–25.5)		(5.5–14.7)		(9.5–18.3)
Quit alcohol	23	9.8	17	8.1	40	9
		(5.1–18.0)		(4.7–13.6)		(5.8–13.7)
Increase physical activity	60	26.7	156	39	216	32.5
		(19.5–35.4)		(31.2–47.4)		(27.2–38.4)
Control weight	45	18.4	100	23.2	145	20.6
		(11.8–27.6)		(17.5–30.1)		(16.0–26.2)
Reduce salt intake	101	45.4	179	37.3	280	41.6
		(36.1–55.1)		(28.7–46.7)		(35.0–48.4)
Modify diet	116	49.7	206	49.7	322	49.7
		(41.2–58.3)		(39.7–59.7)		(43.1–56.3)
Practice Yoga	28	13.9	54	11.2	82	12.6
		(8.6–21.8)		(7.5–16.4)		(9.1–17.2)

^a^Three in rural and four in urban did not report any source of medicines.

^b^Adherence to treatment for hypertension in last 2 weeks.

^c^Advised by health care provider in the last 1 year to hypertensive patients.

Among the hypertensives currently on treatment, those being in the age group 50–69 years (aOR 0.47; 95% CI: 0.26–0.84) and current smokeless tobacco users (aOR 0.31; 95% CI: 0.15–0.64) were less likely to have their BP controlled. Whereas those in the rural areas (aOR 2.19; 95% CI: 1.25–3.83) and those underweight (aOR 4.87; 95% CI: 1.52–15.65) were more likely to have their BP controlled (Table 4).

**Table 4:** Determinants of control of Hypertension (for those who are on treatment).

	*n*	Not controlled %	Controlled %	Crude OR	Adjusted OR
		(95% CI)	(95% CI)	(95% CI)	(95% CI)
Age group (in years)					
30–49	135	47.8	52.2	1	1
		(35.0–60.8)	(39.2–65.0)		
18–29	22	33.9	66.1	1.69	0.81
		(9.3–71.8)	(28.2–90.7)	(0.31–9.16)	(0.18–3.73)
50–69	281	61.8	38.2	0.54	**0.47**
		(54.8–68.4)	(31.6–45.2)	(0.30–0.95)	**(0.26–0.84)**
Place of residence					
Urban	208	47.2	52.8	1	1
		(37.1–57.5)	(42.5–62.9)		
Rural	230	49.2	50.8	2.1	**2.19**
		(37.2–61.4)	(38.6–62.8)	(1.24–3.57)	**(1.25–3.83)**
Sex					
Female	259	60.8	39.2	1	1
		(52.8–68.3)	(31.7–47.2)		
Male	179	49.2	50.8	1.56	1.72
		(37.2–61.4)	(38.6–62.8)	(0.86–2.84)	(0.93–3.18)
Body mass index					
Normal	160	58.7	41.3	1	1
		(47.3–69.2)	(30.8–52.7)		
Underweight	21	26	74	4.05	**4.87**
		(11.1–49.7)	(50.3–88.9)	(1.31–12.55)	**(1.52–15.65)**
Overweight	186	56.5	43.5	1.09	1.39
		(45.2–67.2)	(32.8–54.8)	(0.61–1.97)	(0.68–2.81)
Obese	63	54.3	45.7	1.19	2.06
		(40.7–67.3)	(32.7–59.3)	(0.58–2.46)	(0.81–5.26)
Waist circumference					
Not Raised	150	51.3	48.7	1	1
		(38.9–63.5)	(36.5–61.1)		
Raised	282	58.2	41.8	0.76	0.81
		(50.9–65.2)	(34.8–49.1)	(0.44–1.30)	(0.39–1.69)
Physical activity					
Adequate	195	56.6	43.4	1	1
		(46.5–66.2)	(33.8–53.5)		
Inadequate	243	55.7	44.3	1.06	1.57
		(47.0–64.0)	(36.0–53.0)	(0.64–1.74)	(0.98–2.52)
Current smoked tobacco use					
Non-current	397	58.3	41.7	1	1
		(51.5–64.8)	(35.2–48.5)		
Current	41	34.4	65.6	2.62	1.51
		(15.1–60.8)	(39.2–84.9)	(0.87–7.85)	(0.44–5.15)
Current smokeless tobacco use					
Non-current	379	53.4	46.6	1	1
		(45.9–60.7)	(39.3–54.1)		
Current	59	73.4	26.6	0.41	**0.31**
		(58.3–84.4)	(15.6–41.7)	(0.20–0.85)	**(0.15–0.64)**
Hazardous drinking					
No	423	57	43	1	1
		(50.2–63.6)	(36.4–49.8)		
Yes	15	30.2	69.8	3.01	1.36
		(7.7–69.1)	(30.9–92.3)	(0.59–15.37)	(0.24–7.59)
Dietary salt related practices					
At least one^a^ measure taken on regularly	263	57	43	1	1
		(48.7–64.9)	(35.1–51.3)		
None	175	54.8	45.2	1.13	1.43
		(44.5–64.7)	(35.3–55.5)	(0.71–1.81)	(0.87–2.35)
Co-morbidities^b^					
No other self-reported chronic disease	264	54.2	45.8	1	1
		(45.8–62.3)	(37.7–54.2)		
Yes	174	59	41	0.81	1.1
		(49.2–68.1)	(31.9–50.8)	(0.51–1.27)	(0.63–1.92)
Adherence^c^					
≥11 days	309	61.4	38.6	1	1
		(54.1–68.2)	(31.8–45.9)		
6–10 days	81	45.3	54.7	2.34	1.3
		(28.1–63.7)	(36.3–71.9)	(1.04–5.25)	(0.62–2.73)
≤5 days	48	39.9	60.1	1.88	1.56
		(23.6–58.8)	(41.2–76.4)	(0.86–4.15)	(0.62–3.93)
Physicians consulted for Hypertension					
Govt. health facility	96	50.8	49.2	1	1
		(38.2–63.2)	(36.8–61.8)		
PVT/NGO health facility	302	56.9	43.1	0.78	0.9
		(47.7–65.7)	(34.3–52.3)	(0.41–1.48)	(0.37–2.19)
No	39	62.6	37.4	0.63	0.49
		(43.9–78.2)	(21.8–56.1)	(0.25–1.56)	(0.17–1.41)
Source of medicines					
Govt. facility	79	49.6	50.4	1	1
		(36.9–62.3)	(37.7–63.1)		
Chemist/Private/NGO Dispensary	344	56.7	43.3	0.75	0.71
		(48.5–64.7)	(35.3–51.5)	(0.41–1.38)	(0.30–1.69)

Bold values indicate statistical significance *p* < 0.05.

^a^Measures to control salt intake: limit consumption of high salt containing food/Look at the salt or sodium content on food labels/use of low salt or sodium alternatives/Use spices other than salt/avoid foods prepared outside home/other measures.

^b^Diabetes/hypercholesterolemia/CVD.

^c^Adherence to treatment in last 2 weeks.

Sub-group analysis based on gender (male and female) and area of residence (rural and urban) was carried out for ever measured BP, prevalence of high BP, its awareness, treatment and control status was presented as Supplementary Tables 1a–d and 2a–d. In addition, determinants for control status among those who are under treatment for high BP was depicted in Supplementary Table 4a–d. Age group, level of education and wealth index was found to be significant determinants for prevalence, awareness and treatment of high BP.

## Discussion

This nationally representative study from India found low levels of awareness, treatment, and control of hypertension among adults. In the hypertension cascade, the biggest drops were seen at the awareness and treatment stage. Only 28% of those with high BP were aware of it and 52% of those aware were on treatment. Most of the patients with hypertension on treatment had good adherence and were under control. Key differentials by rural-urban, wealth index and geographical regions were noted.

Women were more likely to have their BP measured (perhaps owing to ante-natal care services) as compared to men. However, the probability of treatment and control did not vary by gender. Mohanty et al., based on an analysis of the NFHS 2015-16, reported that women above 45 years had higher levels of awareness, treatment, and control of hypertension than their male counterparts [19]. Other surveys have also confirmed this [11, 13]. While not statistically significant, our study reported higher treatment rates among women and lower control rates, maybe due to the lower age profile of the participants.

The study highlighted the socioeconomic patterning of the disease. The gap between the lowest and highest wealth quintile was larger for awareness (17.3% vs. 41.8%) and narrower for control (9.4% vs. 16.6%). However, neither treatment nor control of hypertension was determined by the wealth index. Other studies have confirmed the pro-rich inequalities in hypertension awareness, treatment and control [11, 13, 19]. In pooled data from nationally representative studies in low-income and middle-income countries (LMICs), being a woman, older, more educated, wealthier, and not being a current smoker were all positively associated with attaining each of the four steps of the care cascade [20].

The regional and rural-urban differences have been reported from many previous studies [6, 11, 13]. These reflect the differences in the prevalence of different behavioral, risk factors like tobacco use and diet, urbanization levels, socioeconomic differences as well as the organization of health services. Regional differences in the international context have also been reported globally with Latin American and Caribbean countries doing better than sub-Saharan Africa and Asia [20]. These reviews show that improvements in the detection, treatment and control of hypertension have varied substantially across countries, with some middle-income countries now outperforming most high-income nations [20, 21].

Control of BP is influenced both by the population and patient-level factors as well as limitations within the healthcare system [22]. Weak health systems have been identified as major bottlenecks in effectively responding to the rising burden of chronic conditions in LMIC, including India [23]. India has a mixed health care system, with a predominant share by the private sector which is largely unregulated and poorly coordinated, which have posed significant challenges in addressing chronic NCDs adequately [24, 25]. Health facility component of the current study (NNMS)) has well-documented significant gaps in both private and public primary care facilities and public secondary facilities in the availability of essential medicines, technologies, training of available manpower and counseling services of the health system response to NCDs in India. Our efforts have focused on strengthening primary and secondary public health facilities by improving the availability of key medicines for hypertension [26]. The India Hypertension Control Initiative [27], has demonstrated a substantial improvement of BP controls through five intervention strategies of protocol-based approach, ensuring drug availability of a small list of drugs, patient-centric care provision and cohort monitoring enabled by the use of technology of the HEARTS technical package [28]. Currently, this initiative does not include the private sector and an exploratory study involving private practitioners as a part of IHCI in Bhopal (Central part of India) showed that there were major constraints in terms of limited availability of single component hypertension drugs, preferences for fixed-dose combinations, and fear of losing patients to others. In addition, none of the interviewed doctors had resources to provide patient-centered care and use a digital health information system [29] It will be important to address these challenges if we want to achieve better population control of hypertension.

This study provides valuable insight for strategizing for the same and a good template to monitor progress in this regard. Strengths and limitations: The strengths of the study are its national scope, focus on NCD risk factors, good quality assurance, use of standard definitions, large sample size, weighted proportions, high response rates as well as coverage of age group used for global monitoring and linkage to national efforts. Limitations were a single day measurement of BP and adherence to treatment being based on reported medication intake and not on any standard tool or pill counting methods. Other limitations of the survey were challenges in arriving at State-based estimates, since the study sample was nationally representative in line with the specific objectives for generating baseline evidence on risk factors and health-seeking behaviors as per the National NCD Monitoring Framework for India.

## Summary table

### What is already known

- Population-level hypertension control is poor in many developed as well as developing countries.
- Urban-rural, gender, poor-rich differentials exist in hypertension control cascade.

### What this study adds to existing knowledge

- Poor control of Hypertension in India is mainly due to poor awareness of hypertension and inadequate treatment-seeking.
- There exists gender, economic and regional differentials in awareness, treatment and control of hypertension.
- Only 1 in 5 persons with hypertension seeks treatment in the public sector.
- The study identifies population-based screening and health system strengthening including involvement of the private sector as key interventions to improve population-level control of hypertension.

## Supplementary information

Supplementary files: Figure 1a, Figure 1b, Table 1a, Table 1b, Table 1c, Table 1d, Table 2a, Table 2b, Table 2c, Table 2d, Table 4a, Table 4b, Table 4c, Table 4d

## Data Availability

All the data are available within the manuscript. The National Noncommunicable Disease Monitoring Survey (NNMS) report is available at https://www.ncdirindia.org/nnms/.
