# Correction to: Hypertension treatment cascade in India: results from National Noncommunicable Disease Monitoring Survey

**DOI:** 10.1038/s41371-022-00727-4

**Published:** 2022-08-02

**Authors:** Ritvik Amarchand, Vaitheeswaran Kulothungan, Anand Krishnan, Prashant Mathur

**Affiliations:** 1 Centre for Community Medicine, All India Institute of Medical Sciences, New Delhi, India; 2 Indian Council Medical Research (ICMR)—National Centre for Disease Informatics and Research (NCDIR), Bengaluru, Karnataka India

**Keywords:** Risk factors, Hypertension

Correction to: *Journal of Human Hypertension* 10.1038/s41371-022-00692-y, published online 05 May 2022

The original version of this article contained a mistake. Owing to a typesetting error, the definition of High blood pressure missed the following “or who reported having been diagnosed with hypertension by a health professional” in the published article.

The correct definition of High blood pressure is mentioned below.

High BP was defined as systolic blood pressure (SBP) ≥140 mmHg or diastolic blood pressure (DBP) ≥90 mmHg (based on the mean of the 2nd and 3rd measurements of BP based on the minimum percentage regression to mean (SBP: 5.8% and DBP: 9.9%) compared to other two measurement combination) or the participants who reported being currently on medications for raised BP or who reported having been diagnosed with hypertension by a health professional.

The original article has been corrected.

