# Supplementary material for: Hypertension treatment cascade in India: results from National Noncommunicable Disease Monitoring Survey
Source: J Hum Hypertens. 2022 May 5;37(5):394–404. doi: 10.1038/s41371-022-00692-y (PMC10156594; doi:10.1038/s41371-022-00692-y)
Supplement: Supplementary file 1 — Supplementary files: Figure 1a, Figure 1b, Table 1a, Table 1b, Table 1c, Table 1d, Table 2a, Table 2b, Table 2c, Table 2d, Table 4a, Table 4b, Table 4c, Table 4d [file 41371_2022_692_MOESM1_ESM.docx]

**SUPPLEMENTARY FIGURE LEGENDS**

1. Supplementary Fig. 1a: Clusters showing the mapping of National NCD Monitoring Survey sample coverage
2. Supplementary Fig. 1b: Number of adults included for hypertension analysis in the survey

**SUPPLEMENTARY TABLES LEGENDS**

1. Supplementary Table 1a: Measurement of Blood Pressure and Prevalence of high blood pressure and their determinants among male population of India
2. Supplementary Table 1b: Measurement of Blood Pressure and Prevalence of high blood pressure and their determinants among female population of India
3. Supplementary Table 1c: Measurement of Blood Pressure and Prevalence of high blood pressure and their determinants among urban population of India
4. Supplementary Table 1d: Measurement of Blood Pressure and Prevalence of high blood pressure and their determinants among rural population of India
5. Supplementary Table 2a: Prevalence (%) and determinants (aOR with 95% CI) of awareness, treatment, and control among those with hypertension for males
6. Supplementary Table 2b: Prevalence (%) and determinants (aOR with 95% CI) of awareness, treatment, and control among those with hypertension for females
7. Supplementary Table 2c: Prevalence (%) and determinants (aOR with 95% CI) of awareness, treatment, and control among those with hypertension in urban areas
8. Supplementary Table 2d: Prevalence (%) and determinants (aOR with 95% CI) of awareness, treatment, and control among those with hypertension in rural areas
9. Supplementary Table 4a: Determinants of control of Hypertension (for those who are on treatment) among male population of India
10. Supplementary Table 4b: Determinants of control of Hypertension (for those who are on treatment) among female population of India
11. Supplementary Table 4c: Determinants of control of Hypertension (for those who are on treatment) among urban population of India
12. Supplementary Table 4d: Determinants of control of Hypertension (for those who are on treatment) among rural population of India


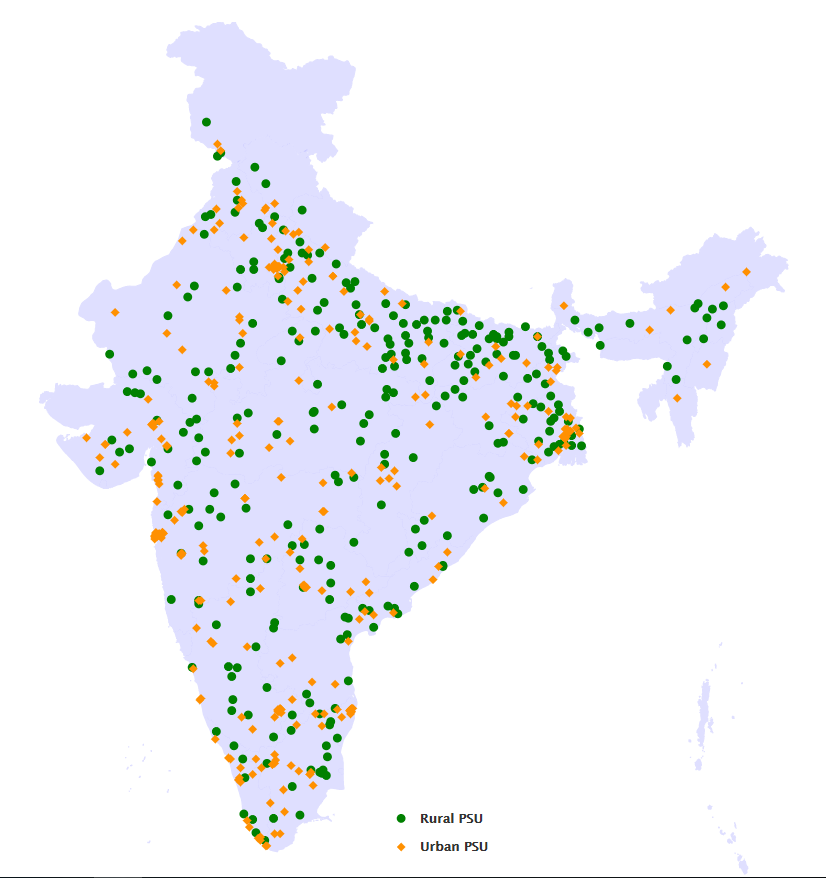


**Supplementary Fig. 1a: Clusters showing the mapping of National NCD Monitoring Survey sample coverage**

National sample - **12000 households**

Household questionnaire completed **- 11139**

**11068 adults participated**

71 adults - Refused to participate / households were locked / selected adult was not available.

409 adults refused to talk and midway refusal.

**10659 adults completed** both adult and household questionnaire **(96.3%)**

One adult per household selected

861 households – Didn’t participate (Door lock/ consent refused) or didn’t complete (Partially complete/midway refusal).

66 adults - Refused to participate / households were locked / selected adult was not available to measure blood pressure value.

**5490 out of 5539 males (99.1%)**

**5103 out of 5120 females (99.7%)**

**3538 out of 3570 Urban adults (99.1%)**

**7055 out of 7089 rural adults (99.5%)**

**10593 adults participated for blood pressure measurement (99.4%)**

**Supplementary Fig. 1b: Number of adults included for hypertension analysis in the survey**

| **Supplementary Table 1a**: **Measurement of Blood Pressure and Prevalence of high blood pressure and their determinants among male population of India** | | | | | | | | |
| --- | --- | --- | --- | --- | --- | --- | --- | --- |
| **Subgroups** | **Ever measured blood pressure** | | | | **High Blood pressure** | | | |
|  | **n** | **Prevalence (%)** | **UOR** | **AOR** | | **Prevalence (%)** | **UOR** | **AOR** |
|  |  | **(95% CI)** | **(95% CI)** | **(95% CI)** | | **(95% CI)** | **(95% CI)** | **(95% CI)** |
| **Total** | 5490 | 40.9 |  |  | | 29.9 |  |  |
|  |  | (38.0 - 43.8) |  |  | | (27.9 - 32.0) |  |  |
| **Age groups (in years)** |  |  |  |  | |  |  |  |
| 18-29 | 1657 | 26.3 | 1 | 1 | | 15.3 | 1 | 1 |
|  |  | (22.6-30.5) |  |  |  | (12.8-18.2) |  |  |
| 30-49 | 2659 | 41.9 | **2.02** | **2.45** | | 31.3 | **2.52** | **2.40** |
|  |  | (38.5-45.4) | **(1.64-2.48)** | **(1.91-3.13)** | | (28.7-34.0) | **(2.00-3.17)** | **(1.87-3.08)** |
| 50-69 | 1174 | 59.0 | **4.03** | **5.19** | | 47.4 | **4.98** | **4.98** |
|  |  | (54.2-63.6) | **(3.10-5.25)** | **(3.84-7.02)** | | (43.3-51.5) | **(3.83-6.48)** | **(3.75-6.62)** |
| **Education*** |  |  |  |  | |  |  |  |
| Primary | 1900 | 31.4 | 1 | 1 | | 29.5 | 1 | 1 |
|  |  | (27.1-36.0) |  |  |  | (26.5-32.7) |  |  |
| Secondary | 1982 | 42.4 | **1.61** | **1.56** | | 29.1 | 0.98 | 1.05 |
|  |  | (38.7-46.2) | **(1.28-2.02)** | **(1.23-1.97)** | | (25.9-32.4) | (0.81-1.18) | (0.84-1.31) |
| Higher secondary and above | 1601 | 50.2 | **2.20** | **1.54** | | 31.3 | 1.09 | 1.18 |
|  |  | (46.0-54.4) | **(1.71-2.83)** | **(1.14-2.08)** | | (28.3-34.5) | (0.88-1.34) | (0.89-1.37) |
| **Occupation** |  |  |  |  | |  |  |  |
| Skilled/unskilled labourers | 2596 | 31.5 | 1 | 1 | | 27.0 | 1 | 1 |
|  |  | (28.2-35.0) |  |  |  | (24.2-29.9) |  |  |
| Not working** | 872 | 45.2 | **1.79** | **1.63** | | 27.1 | 1.01 | 0.94 |
|  |  | (39.2-51.2) | **(1.37-2.33)** | **(1.22-2.18)** | | (22.8-31.9) | (0.78-1.29) | (0.71-1.24) |
| Others*** | 2014 | 51.0 | **2.26** | **1.40** | | 35.0 | **1.46** | 1.11 |
|  |  | (46.8-55.3) | **(1.84-2.79)** | **(1.11-1.77)** | | (31.7-38.3) | **(1.19-1.78)** | (0.89-1.57) |
| **Wealth index Quintiles** |  |  |  |  | |  |  |  |
| Q1 | 1386 | 23.3 | 1 | 1 | | 24.4 | 1 | 1 |
|  |  | (18.8-28.5) |  |  |  | (21.2-28.0) |  |  |
| Q2 | 1282 | 35.4 | **1.80** | **1.67** | | 26.2 | 1.10 | 1.07 |
|  |  | (31.0-39.9) | **(1.37-2.37)** | **(1.25-2.21)** | | (22.6-30.1) | (0.85-1.41) | (0.83-1.38) |
| Q3 | 1021 | 40.6 | **2.26** | **1.74** | | 28.3 | 1.22 | 1.10 |
|  |  | (36.2-45.3) | **(1.66-3.07)** | **(1.25-2.41)** | | (24.3-32.8) | (0.94-1.41) | (0.82-1.48) |
| Q4 | 922 | 50.6 | **3.38** | **2.54** | | 36.9 | **1.81** | **1.41** |
|  |  | (45.3-55.9) | **(2.40-4.76)** | **(1.74-3.69)** | | (32.1-42.0) | **(1.39-2.36)** | **(1.04-1.93)** |
| Q5 | 879 | 66.7 | **6.60** | **4.86** | | 38.5 | 1.93 | 1.25 |
|  |  | (61.3-71.6) | **(4.65-9.36)** | **(3.09-7.64)** | | (34.7-42.3) | (1.53-2.44) | (0.90-1.73) |
| **Regions of India** |  |  |  |  | |  |  |  |
| Central | 1396 | 33.5 | 1 | 1 | | 24.2 | 1 | 1 |
|  |  | (28.8-38.4) |  |  |  | (20.7-28.0) |  |  |
| East | 1176 | 29.7 | 0.84 | 1.03 | | 26.8 | 1.15 | 1.23 |
|  |  | (24.9-34.9) | (0.61-1.16) | (0.76-1.40) | | (21.5-32.8) | (0.81-1.63) | (0.84-1.78) |
| North | 685 | 38.7 | 1.26 | 0.84 | | 38.1 | **1.93** | **1.79** |
|  |  | (32.1-45.8) | (0.87-1.81) | (0.61-1.16) | | (33.1-43.4) | **(1.43-2.61)** | **(1.34-2.38)** |
| South | 1274 | 63.0 | **3.38** | **3.15** | | 35.0 | **1.69** | **1.39** |
|  |  | (57.0-68.6) | **(2.42-4.71)** | **(2.24-4.43)** | | (31.1-39.1) | **(1.29-2.21)** | **(1.09-1.77)** |
| West | 682 | 35.5 | 1.10 | 0.93 | | 28.3 | 1.24 | 1.13 |
|  |  | (30.7-40.7) | (0.80-1.49) | (0.69-1.25) | | (23.6-33.5) | (0.90-1.70) | (0.84-1.52) |
| North-East | 278 | 42.3 | 1.46 | 1.08 | | 32.5 | **1.51** | 1.26 |
|  |  | (32.6-52.7) | (0.91-2.34) | (0.78-1.50) | | (25.7-40.1) | **(1.02-2.23)** | (0.87-1.82) |
| **Place of residence** |  |  |  |  | |  |  |  |
| Rural | 3611 | 34.1 | 1 | 1 | | 26.0 | 1 | 1 |
|  |  | (30.9-37.3) |  |  |  | (23.5-28.8) |  |  |
| Urban | 1879 | 53.9 | **2.27** | 0.99 | | 37.4 | **1.70** | **1.38** |
|  |  | (49.0-58.8) | **(1.78-2.90)** | (0.75-1.29) | | (34.5-40.3) | **(1.41-2.04)** | **(1.11-1.71)** |
| *Primary: illiterate and <6th standard; Secondary: 6th to 10th standard; Higher secondary and above: 11th standard and above | | | | | | | | |
| **Homemaker/ retired/unemployed | | | | | | | | |
| ***professionals /managers/executives /self-employed | | | | | | | | |

| **Supplementary Table 1b**: **Measurement of Blood Pressure and Prevalence of high blood pressure and their determinants among female population of India** | | | | | | | |
| --- | --- | --- | --- | --- | --- | --- | --- |
| **Subgroups** | **Ever measured blood pressure** | | | | **High Blood pressure** | | |
|  | **n** | **Prevalence (%)** | **UOR** | **AOR** | **Prevalence (%)** | **UOR** | **AOR** |
|  |  | **(95% CI)** | **(95% CI)** | **(95% CI)** | **(95% CI)** | **(95% CI)** | **(95% CI)** |
| **Total** | 5103 | 54.8 |  |  | 27.0 |  |  |
|  |  | (51.9 - 57.6) |  |  | (25.2 - 28.8) |  |  |
| **Age groups (in years)** |  |  |  |  |  |  |  |
| 18-29 | 1468 | 47.3 | 1 | 1 | 10.8 | 1 | 1 |
|  |  | (43.5-51.1) |  |  | (8.8-13.2) |  |  |
| 30-49 | 2462 | 55.0 | **1.36** | **1.32** | 24.4 | **2.66** | **2.31** |
|  |  | (51.6-58.4) | **(1.15-1.61)** | **(1.08-1.60)** | (22.0-26.9) | **(2.06-3.45)** | **(1.76-3.02)** |
| 50-69 | 1173 | 63.7 | **1.95** | **1.96** | 52.6 | **9.19** | **7.23** |
|  |  | (59.3-67.9) | **(1.59-2.39)** | **(1.54-2.48)** | (49.2-56.1) | **(7.10-11.89)** | **(5.44-9.60)** |
| **Education*** |  |  |  |  |  |  |  |
| Primary | 2897 | 48.7 | 1 | 1 | 31.9 | 1 | 1 |
|  |  | (45.3-52.2) |  |  | (29.3-34.5) |  |  |
| Secondary | 1368 | 62.6 | **1.76** | **1.40** | 22.4 | **0.62** | **0.78** |
|  |  | (58.9-66.1) | **(1.49-2.08)** | **(1.14-1.71)** | (19.6-25.5) | **(0.51-0.75)** | **(0.62-0.97)** |
| Higher secondary and above | 834 | 63.0 | **1.79** | 1.07 | 17.3 | **0.45** | **0.55** |
|  |  | (57.7-68.0) | **(1.40-2.29)** | (0.81-1.42) | (14.5-20.5) | **(0.35-0.57)** | **(0.40-0.77)** |
| **Occupation** |  |  |  |  |  |  |  |
| Skilled/unskilled labourers | 1009 | 50.2 | 1 | 1 | 27.2 | 1 | 1 |
|  |  | (45.1-55.2) |  |  | (23.4-31.4) |  |  |
| Not working** | 3737 | 55.3 | **1.23** | 1.10 | 26.8 | 0.98 | 1.07 |
|  |  | (52.1-58.4) | **(1.00-1.51)** | (0.87-1.38) | (24.9-28.8) | (0.79-1.22) | (0.85-1.36) |
| Others*** | 354 | 63.3 | **1.71** | 1.01 | 28.1 | 1.04 | 1.10 |
|  |  | (55.0-70.8) | **(1.16-2.50)** | (0.67-1.52) | (22.0-35.1) | (0.70-1.55) | (0.70-1.72) |
| **Wealth index Quintiles** |  |  |  |  |  |  |  |
| Q1 | 1429 | 36.3 | 1 | 1 | 26.9 | 1 | 1 |
|  |  | (32.6-40.2) |  |  | (23.4-30.7) |  |  |
| Q2 | 1138 | 51.8 | **1.88** | **1.73** | 25.7 | 0.94 | 1.06 |
|  |  | (47.3-56.3) | **(1.52-2.33)** | **(1.41-2.13)** | (22.2-29.6) | (0.73-1.20) | (0.82-1.37) |
| Q3 | 1041 | 57.5 | **2.37** | **2.02** | 23.1 | 0.81 | 0.88 |
|  |  | (53.2-61.7) | **(1.91-2.95)** | **(1.61-2.54)** | (20.0-26.5) | (0.63-1.05) | (0.68-1.15) |
| Q4 | 837 | 67.9 | **3.71** | **2.86** | 27.7 | 1.04 | 1.06 |
|  |  | (63.5-72.1) | **(2.89-4.77)** | **(2.20-3.72)** | (24.3-31.5) | (0.80-1.36) | (0.78-1.44) |
| Q5 | 658 | 79.0 | **6.60** | **4.83** | 34.4 | **1.42** | **1.54** |
|  |  | (74.5-83.0) | **(4.88-8.93)** | **(3.41-6.83)** | (30.6-38.3) | **(1.11-1.82)** | **(1.13-2.10)** |
| **Regions of India** |  |  |  |  |  |  |  |
| Central | 1268 | 46.7 | 1 | 1 | 21.8 | 1 | 1 |
|  |  | (40.9-52.5) |  |  | (18.4-25.6) |  |  |
| East | 1167 | 44.1 | 0.90 | 0.99 | 27.4 | **1.35** | 1.30 |
|  |  | (38.8-49.5) | (0.65-1.24) | (0.72-1.35) | (23.7-31.4) | **(1.01-1.80)** | (0.96-1.77) |
| North | 706 | 63.0 | **1.95** | 1.35 | 29.4 | **1.50** | 1.29 |
|  |  | (54.5-70.8) | **(1.27-2.97)** | (0.94-1.62) | (25.3-33.9) | **(1.11-2.01)** | (0.93-1.79) |
| South | 1263 | 67.7 | **2.39** | **1.86** | 32.3 | **1.71** | **1.39** |
|  |  | (63.2-71.9) | **(1.75-3.26)** | **(1.37-2.52)** | (29.2-35.6) | **(1.32-2.21)** | **(1.04-1.86)** |
| West | 494 | 54.7 | 1.38 | 1.08 | 22.8 | 1.06 | 0.99 |
|  |  | (47.4-61.7) | (0.95-2.00) | (0.73-1.60) | (18.5-27.7) | (0.75-1.48) | (0.67-1.47) |
| North-East | 205 | 59.0 | **1.64** | 1.26 | 25.2 | 1.21 | 1.16 |
|  |  | (48.1-69.1) | **(1.00-2.71)** | (0.80-1.98) | (17.9-34.1) | (0.75-1.95) | (0.69-1.94) |
| **Place of residence** |  |  |  |  |  |  |  |
| Rural | 3444 | 49.1 | 1 | 1 | 25.4 | 1 | 1 |
|  |  | (45.4-52.8) |  |  | (23.2-27.6) |  |  |
| Urban | 1659 | 66.7 | **2.07** | 1.11 | 30.2 | **1.27** | 1.21 |
|  |  | (62.9-70.2) | **(1.66-2.58)** | (0.90-1.36) | (27.5-33.0) | **(1.07-1.52)** | (0.99-1.47) |
| *Primary: illiterate and <6th standard; Secondary: 6th to 10th standard; Higher secondary and above: 11th standard and above | | | | | | | |
| **Homemaker/ retired/unemployed | | | | | | | |
| ***professionals /managers/executives /self-employed | | | | | | | |

| **Supplementary Table 1c**: **Measurement of Blood Pressure and Prevalence of high blood pressure and their determinants among urban population of India** | | | | | | | |
| --- | --- | --- | --- | --- | --- | --- | --- |
| **Subgroups** | **Ever measured blood pressure** | | | | **High Blood pressure** | | |
|  | **n** | **Prevalence (%)** | **UOR** | **AOR** | **Prevalence (%)** | **UOR** | **AOR** |
|  |  | **(95% CI)** | **(95% CI)** | **(95% CI)** | **(95% CI)** | **(95% CI)** | **(95% CI)** |
| **Total** | 3538 | 59.9 |  |  | 34.0 |  |  |
|  |  | (56.5 - 63.2) |  |  | (32.0 - 36.1) |  |  |
| **Age groups (in years)** |  |  |  |  |  |  |  |
| 18-29 | 888 | 41.5 | 1 | 1 | 15.2 | 1 | 1 |
|  |  | (36.8-46.4) |  |  | (12.6-18.1) |  |  |
| 30-49 | 1823 | 60.9 | **2.19** | **2.32** | 32.7 | **2.72** | **2.48** |
|  |  | (57.2-64.5) | **(1.75-2.74)** | **(1.80-2.99)** | (29.5-36.1) | **(2.01-3.67)** | **(1.82-3.38)** |
| 50-69 | 827 | 77.4 | **4.83** | **5.09** | 57.1 | **7.44** | **6.80** |
|  |  | (72.1-82.0) | **(3.54-6.58)** | **(3.76-6.90)** | (52.1-62.0) | **(5.44-10.18)** | **(4.83-9.56)** |
| **Sex** |  |  |  |  |  |  |  |
| Male | 1879 | 53.9 | 1 | 1 | 37.4 | 1 | 1 |
|  |  | (49.0-58.8) |  |  | (34.5-40.3) |  |  |
| Female | 1659 | 66.7 | **1.71** | **1.71** | 30.2 | **0.73** | **0.69** |
|  |  | (62.9-70.2) | **(1.35-2.16)** | **(1.17-2.49)** | (27.5-33.0) | **(0.61-0.86)** | **(0.56-0.85)** |
| **Education*** |  |  |  |  |  |  |  |
| Primary | 967 | 56.6 | 1 | 1 | 40.8 | 1 | 1 |
|  |  | (52.0-61.1) |  |  | (36.8-45.0) |  |  |
| Secondary | 1212 | 60.3 | 1.16 | 1.16 | 33.2 | **0.72** | **0.75** |
|  |  | (55.7-64.7) | (0.93-1.45) | (0.89-1.50) | (29.5-37.1) | **(0.58-0.90)** | **(0.60-0.95)** |
| Higher secondary and above | 1354 | 62.0 | **1.25** | 1.17 | 29.9 | **0.62** | **0.68** |
|  |  | (57.6-66.2) | **(1.01-1.54)** | (0.87-1.57) | (27.3-32.7) | **(0.49-0.78)** | **(0.51-0.91)** |
| **Occupation** |  |  |  |  |  |  |  |
| Skilled/unskilled labourers | 858 | 49.6 | 1 | 1 | 36.1 | 1 | 1 |
|  |  | (44.8-54.3) |  |  | (31.3-41.1) |  |  |
| Not working** | 1581 | 64.5 | **1.85** | 1.41 | 30.2 | **0.77** | 0.90 |
|  |  | (60.4-68.4) | **(1.47-2.33)** | (0.94-2.11) | (27.4-33.2) | **(0.60-0.99)** | (0.67-1.21) |
| Others*** | 1097 | 61.4 | **1.62** | 1.23 | 37.9 | 1.08 | 1.07 |
|  |  | (56.2-66.3) | **(1.28-2.04)** | (0.90-1.69) | (33.7-42.2) | (0.81-1.45) | (0.78-1.47) |
| **Wealth index Quintiles** |  |  |  |  |  |  |  |
| Q1 | 312 | 44.5 | 1 | 1 | 37.5 | 1 | 1 |
|  |  | (34.7-54.8) |  |  | (28.8-47.0) |  |  |
| Q2 | 498 | 51.9 | 1.35 | **1.59** | 34.0 | 0.86 | 0.94 |
|  |  | (44.5-59.2) | (0.98-1.84) | **(1.12-2.26)** | (27.7-40.9) | (0.58-1.28) | (0.61-1.44) |
| Q3 | 646 | 51.0 | 1.30 | **1.61** | 28.4 | 0.66 | 0.78 |
|  |  | (45.8-56.2) | (0.85-1.97) | **(1.05-2.45)** | (24.0-33.4) | (0.41-1.08) | (0.46-1.32) |
| Q4 | 918 | 60.4 | **1.90** | **2.21** | 33.6 | 0.84 | 0.99 |
|  |  | (55.8-64.8) | **(1.22-2.96)** | **(1.42-3.43)** | (29.4-38.0) | (0.54-1.31) | (0.63-1.56) |
| Q5 | 1165 | 72.0 | **3.21** | **3.94** | 36.6 | 0.96 | 1.08 |
|  |  | (67.8-75.8) | **(2.07-4.95)** | **(2.52-6.16)** | (33.6-39.6) | (0.64-1.45) | (0.67-1.73) |
| **Regions of India** |  |  |  |  |  |  |  |
| Central | 782 | 54.7 | 1 | 1 | 30.7 | 1 | 1 |
|  |  | (49.0-60.3) |  |  | (26.9-34.8) |  |  |
| East | 439 | 45.0 | **0.68** | **0.75** | 28.9 | 0.92 | **0.85** |
|  |  | (39.0-51.2) | **(0.48-0.95)** | **(0.54-1.05)** | (25.0-33.2) | (0.70-1.21) | **(0.60-1.20)** |
| North | 410 | 58.1 | 1.15 | 0.84 | 39.4 | **1.47** | **1.42** |
|  |  | (49.8-66.0) | (0.76-1.72) | (0.57-1.23) | (33.2-46.0) | **(1.06-2.04)** | **(1.01-1.99)** |
| South | 1331 | 70.2 | **1.95** | **2.05** | 36.5 | **1.30** | 1.13 |
|  |  | (64.0-75.7) | **(1.35-2.80)** | **(1.42-2.96)** | (33.0-40.2) | **(1.01-1.66)** | (0.86-1.47) |
| West | 404 | 52.3 | 0.91 | 0.89 | 32.1 | 1.07 | 1.00 |
|  |  | (45.2-59.3) | (0.63-1.31) | (0.59-1.33) | (28.3-36.2) | (0.82-1.39) | (0.73-1.36) |
| North-East | 173 | 64.0 | **1.47** | 1.17 | 34.3 | 1.18 | 1.08 |
|  |  | (60.1-67.8) | **(1.11-1.96)** | (0.73-1.90) | (30.1-38.8) | (0.90-1.54) | (0.73-1.60) |
| *Primary: illiterate and <6th standard; Secondary: 6th to 10th standard; Higher secondary and above: 11th standard and above | | | | | | | |
| **Homemaker/ retired/unemployed | | | | | | | |
| ***professionals /managers/executives /self-employed | | | | | | | |

| **Supplementary Table 1d**: **Measurement of Blood Pressure and Prevalence of high blood pressure and their determinants among rural population of India** | | | | | | | |
| --- | --- | --- | --- | --- | --- | --- | --- |
| **Subgroups** | **Ever measured blood pressure** | | | | **High Blood pressure** | | |
|  | **n** | **Prevalence (%)** | **UOR** | **AOR** | **Prevalence (%)** | **UOR** | **AOR** |
|  |  | **(95% CI)** | **(95% CI)** | **(95% CI)** | **(95% CI)** | **(95% CI)** | **(95% CI)** |
| **Total** | 7055 | 41.4 |  |  | 25.7 |  |  |
|  |  | (38.5 - 44.3) |  |  | (23.8 - 27.8) |  |  |
| **Age groups (in years)** |  |  |  |  |  |  |  |
| 18-29 | 2237 | 34.1 | 1 | 1 | 12.4 | 1 | 1 |
|  |  | (30.7-37.6) |  |  | (10.4-14.8) |  |  |
| 30-49 | 3297 | 41.2 | **1.36** | **1.52** | 25.3 | **2.40** | **2.41** |
|  |  | (37.8-44.7) | **(1.17-1.57)** | **(1.28-1.81)** | (22.9-27.9) | **(1.95-2.94)** | **(1.93-3.00)** |
| 50-69 | 1520 | 52.6 | **2.15** | **2.51** | 46.1 | **6.05** | **5.94** |
|  |  | (48.4-56.8) | **(1.79-2.58)** | **(2.01-3.13)** | (42.8-49.6) | **(4.81-7.60)** | **(4.61-7.65)** |
| **Sex** |  |  |  |  |  |  |  |
| Male | 3611 | 34.1 | 1 | 1 | 26.0 | 1 | 1 |
|  |  | (30.9-37.3) |  |  | (23.5-28.8) |  |  |
| Female | 3444 | 49.1 | **1.87** | **2.04** | 25.4 | 0.97 | 0.92 |
|  |  | (45.4-52.8) | **(1.59-2.19)** | **(1.67-2.51)** | (23.2-27.6) | (0.83-1.12) | (0.76-1.12) |
| **Education*** |  |  |  |  |  |  |  |
| Primary | 3829 | 38.1 | 1 | 1 | 28.4 | 1 | 1 |
|  |  | (34.7-41.7) |  |  | (26.0-31.0) |  |  |
| Secondary | 2138 | 45.1 | **1.33** | **1.56** | 22.5 | **0.73** | 0.96 |
|  |  | (41.4-48.9) | **(1.14-1.56)** | **(1.31-1.87)** | (19.5-25.7) | **(0.61-0.87)** | (0.78-1.18) |
| Higher secondary and above | 1081 | 45.3 | **1.34** | **1.34** | 22.2 | **0.72** | 1.09 |
|  |  | (40.6-50.2) | **(1.07-1.69)** | **(1.00-1.80)** | (18.5-26.4) | **(0.56-0.92)** | (0.82-1.43) |
| **Occupation** |  |  |  |  |  |  |  |
| Skilled/unskilled labourers | 3028 | 47.5 | 1 | 1 | 24.2 | 1 | 1 |
|  |  | (43.9-51.1) |  |  | (21.5-27.1) |  |  |
| Not working** | 2748 | 32.8 | **1.86** | **1.34** | 25.1 | 1.05 | 1.11 |
|  |  | (29.1-36.7) | **(1.54-2.24)** | **(1.09-1.65)** | (22.7-27.6) | (0.89-1.24) | (0.88-1.39) |
| Others*** | 1271 | 45.5 | **1.71** | **1.38** | 30.5 | **1.38** | 1.18 |
|  |  | (40.0-51.1) | **(1.32-2.22)** | **(1.05-1.80)** | (27.0-34.3) | **(1.11-1.71)** | (0.94-1.48) |
| **Wealth index Quintiles** |  |  |  |  |  |  |  |
| Q1 | 2503 | 28.1 | 1 | 1 | 24.2 | 1 | 1 |
|  |  | (25.0-31.3) |  |  | (21.7-27.0) |  |  |
| Q2 | 1923 | 40.8 | **1.77** | **1.66** | 23.9 | 0.98 | 1.04 |
|  |  | (37.2-44.5) | **(1.47-2.12)** | **(1.39-1.98)** | (21.1-26.9) | (0.81-1.19) | (0.85-1.28) |
| Q3 | 1416 | 48.3 | **2.40** | **1.91** | 24.4 | 1.01 | 1.00 |
|  |  | (44.2-52.5) | **(1.96-2.93)** | **(1.55-2.35)** | (20.7-28.6) | (0.79-1.29) | (0.77-1.31) |
| Q4 | 841 | 57.2 | **3.42** | **2.82** | 31.4 | **1.43** | **1.30** |
|  |  | (51.7-62.4) | **(2.61-4.48)** | **(2.14-3.72)** | (27.0-36.3) | **(1.13-1.82)** | **(0.99-1.70)** |
| Q5 | 372 | 71.8 | **6.54** | **5.33** | 37.2 | **1.85** | **1.57** |
|  |  | (63.4-79.0) | **(4.29-9.96)** | **(3.42-8.32)** | (31.9-42.8) | **(1.40-2.44)** | **(1.14-2.14)** |
| **Regions of India** |  |  |  |  |  |  |  |
| Central | 1881 | 33.5 | 1 | 1 | 19.9 | 1 | 1 |
|  |  | (29.3-38.0) |  |  | (16.9-23.2) |  |  |
| East | 1904 | 34.9 | 1.06 | 1.09 | 26.7 | **1.47** | **1.43** |
|  |  | (30.2-40.0) | (0.79-1.43) | (0.81-1.46) | (22.1-31.7) | **(1.07-2.01)** | **(1.04-1.97)** |
| North | 981 | 48.1 | 1.84 | 1.18 | 31.3 | **1.84** | **1.54** |
|  |  | (39.2-57.2) | (1.22-2.78) | (0.85-1.62) | (26.8-36.2) | **(1.37-2.47)** | **(1.12-2.12)** |
| South | 1206 | 59.9 | 2.96 | 2.57 | 30.5 | **1.77** | **1.52** |
|  |  | (55.0-64.7) | (2.24-3.93) | (1.87-3.53) | (26.5-34.7) | **(1.34-2.33)** | **(1.14-2.01)** |
| West | 772 | 39.0 | 1.27 | 1.04 | 22.7 | 1.19 | 1.09 |
|  |  | (32.4-46.1) | (0.89-1.80) | (0.71-1.51) | (18.6-27.5) | (0.86-1.64) | (0.78-1.51) |
| North-East | 310 | 41.2 | 1.39 | 1.18 | 26.6 | **1.46** | 1.28 |
|  |  | (32.9-50.0) | (0.93-2.09) | (0.84-1.65) | (20.9-33.2) | **(1.01-2.13)** | (0.87-1.88) |
| *Primary: illiterate and <6th standard; Secondary: 6th to 10th standard; Higher secondary and above: 11th standard and above | | | | | | | |
| **Homemaker/ retired/unemployed | | | | | | | |
| ***professionals /managers/executives /self-employed | | | | | | | |

| **Supplementary Table 2a**: **Prevalence (%) and determinants (aOR with 95% CI) of awareness, treatment, and control among those with hypertension for males** | | | | | | | | | | | | |
| --- | --- | --- | --- | --- | --- | --- | --- | --- | --- | --- | --- | --- |
| **Subgroups** | **Awareness** | | | | **Treatment** | | | | **Control** | | | |
|  | **n** | **Prevalence (%)** | **UOR** | **AOR** | | **Prevalence (%)** | **UOR** | **AOR** | | **Prevalence (%)** | **UOR** | **AOR** |
|  |  | **(95% CI)** | **(95% CI)** | **(95% CI)** | | **(95% CI)** | **(95% CI)** | **(95% CI)** | | **(95% CI)** | **(95% CI)** | **(95% CI)** |
| **Total** | 1642 | 22.6 |  |  | | 10.9 |  |  | | 10.3 |  |  |
|  |  | (19.8 - 25.7) |  |  |  | (8.9 - 13.3) |  |  |  | (8.2 - 12.8) |  |  |
| **Age groups (in years)** |  |  |  |  | |  |  |  | |  |  |  |
| 18-29 | 254 | 13.9 | 1 | 1 | | 5.3 | 1 | 1 | | 9.3 | 1 | 1 |
|  |  | (8.4-22.1) |  |  |  | (1.8-14.6) |  |  |  | (4.8-17.2) |  |  |
| 30-49 | 832 | 18.7 | 1.43 | 1.67 | | 8.3 | 1.26 | 1.81 | | 9.5 | 0.64 | 0.88 |
|  |  | (14.8-23.4) | (0.73-2.82) | (0.86-3.27) | | (5.4-12.4) | (0.32-4.97) | (0.47-6.94) | | (6.5-13.8) | (0.06-7.03) | (0.13-6.07) |
| 50-69 | 556 | 32.4 | **2.98** | **3.23** | | 17.3 | 1.83 | 2.52 | | 11.9 | 0.31 | 0.34 |
|  |  | (27.1-38.2) | **(1.55-5.72)** | **(1.66-6.30)** | | (13.3-22.1) | (0.47-7.08) | (0.63-10.13) | | (8.5-16.4) | (0.03-3.16) | (0.05-2.23) |
| **Education*** |  |  |  |  | |  |  |  | |  |  |  |
| Primary | 561 | 20.4 | 1 | 1 | | 7.8 | 1 | 1 | | 12.4 | 1 | 1 |
|  |  | (16.3-25.2) |  |  |  | (4.9-12.2) |  |  |  | (8.7-17.2) |  |  |
| Secondary | 576 | 20.1 | 0.98 | 0.89 | | 12.1 | 2.41 | 2.07 | | 7.2 | 0.51 | 0.55 |
|  |  | (15.7-25.3) | (0.66-1.46) | (0.56-1.40) | | (8.4-17.0) | (1.03-5.66) | (0.95-4.51) | | (4.7-10.9) | (0.15-1.70) | (0.15-1.96) |
| Higher secondary and above | 501 | 27.8 | 1.50 | 1.12 | | 13 | 1.41 | 1.40 | | 11.6 | 0.82 | 0.44 |
|  |  | (21.9-34.5) | (0.99-2.28) | (0.63-2.01) | | (8.9-18.6) | (0.62-3.20) | (0.52-3.78) | | (7.2-18.1) | (0.24-2.80) | (0.10-1.89) |
| **Occupation** |  |  |  |  | |  |  |  | |  |  |  |
| Skilled/unskilled labourers | 700 | 14.2 | 1 | 1 | | 5.6 | 1 | 1 | | 7.8 | 1 | 1 |
|  |  | (11.1-18.1) |  |  |  | (3.7-8.3) |  |  |  | (5.5-10.9) |  |  |
| Not working** | 236 | 32.8 | **2.94** | **2.14** | | 18 | 1.90 | 2.06 | | 14 | 1.27 | 2.45 |
|  |  | (25.1-41.6) | **(1.79-4.83)** | **(1.19-3.83)** | | (12.1-26.0) | (0.79-4.54) | (0.84-5.08) | | (8.3-22.8) | (0.37-4.43) | (0.62-9.74) |
| Others*** | 704 | 27.5 | **2.29** | **1.67** | | 13.8 | 1.56 | 1.65 | | 11.6 | 1.19 | 1.12 |
|  |  | (22.5-33.2) | **(1.55-3.39)** | **(1.07-2.61)** | | (9.8-19.1) | (0.73-3.30) | (0.72-3.77) | | (7.9-16.7) | (0.40-3.57) | (0.32-3.92) |
| **Wealth index Quintiles** |  |  |  |  | |  |  |  | |  |  |  |
| Q1 | 339 | 13.8 | 1 | 1 | | 5.3 | 1 | 1 | | 8.1 | 1 | 1 |
|  |  | (9.7-19.3) |  |  |  | (2.7-10.4) |  |  |  | (4.9-13.0) |  |  |
| Q2 | 336 | 21.5 | 1.70 | 1.72 | | 10.6 | 1.56 | 1.94 | | 10.7 | 1.93 | 0.94 |
|  |  | (15.1-29.5) | (0.92-3.16) | (0.91-3.25) | | (5.7-18.8) | (0.49-4.99) | (0.62-6.08) | | (5.7-19.0) | (0.30-12.57) | (0.17-5.16) |
| Q3 | 289 | 17.9 | 1.36 | 1.48 | | 7.8 | 1.22 | 1.20 | | 9.1 | 2.26 | 2.01 |
|  |  | (12.8-24.6) | (0.77-2.42) | (0.80-2.74) | | (4.8-12.3) | (0.40-3.78) | (0.98-3.83) | | (5.7-14.4) | (0.42-12.03) | (0.45-8.97) |
| Q4 | 340 | 24.3 | **2.00** | **1.95** | | 13.2 | 1.87 | 1.62 | | 11.4 | 1.46 | 1.48 |
|  |  | (18.5-31.2) | **(1.15-3.49)** | **(1.06-3.60)** | | (8.8-19.2) | (0.64-5.53) | (0.54-4.82) | | (6.6-19.1) | (0.26-7.99) | (0.28-7.84) |
| Q5 | 338 | 34.8 | **3.33** | **3.33** | | 17.1 | 1.53 | 1.51 | | 12.1 | 1.32 | 2.58 |
|  |  | (27.6-42.7) | **(1.94-5.70)** | **(1.77-6.29)** | | (11.4-24.7) | (0.52-4.50) | (0.44-5.21) | | (7.6-18.7) | (0.25-6.79) | (0.50-13.24) |
| **Regions of India** |  |  |  |  | |  |  |  | |  |  |  |
| Central | 337 | 22.9 | 1 | 1 | | 7.2 | 1 | 1 | | 12.6 | 1 | 1 |
|  |  | (17.6-29.2) |  |  |  | (4.4-11.6) |  |  |  | (8.4-18.4) |  |  |
| East | 315 | 19.6 | 0.82 | 0.94 | | 12.3 | 3.70 | 4.56 | | 13.4 | 1.09 | 0.43 |
|  |  | (13.7-27.3) | (0.48-1.41) | (0.52-1.70) | | (7.7-19.3) | (1.39-5.53) | (1.58-13.21) | | (8.1-21.3) | (0.24-5.04) | (0.05-3.43) |
| North | 261 | 26.4 | 1.21 | 0.91 | | 12.8 | 2.05 | 1.73 | | 10.7 | 0.53 | 0.29 |
|  |  | (18.5-36.2) | (0.69-2.12) | (0.51-1.63) | | (7.5-21.0) | (0.78-5.36) | (0.60-5.03) | | (5.8-18.9) | (0.10-2.87) | (0.04-1.76) |
| South | 446 | 24.9 | 1.11 | 1.08 | | 14.4 | 3.02 | 2.71 | | 8.3 | 0.31 | 0.25 |
|  |  | (19.4-31.2) | (0.71-1.75) | (0.68-1.74) | | (10.5-19.5) | (1.32-6.89) | (1.12-6.56) | | (5.3-12.8) | (0.09-1.09) | (0.05-1.19) |
| West | 193 | 13.8 | 0.54 | 0.53 | | 7.1 | 2.34 | 2.51 | | 6.6 | 0.58 | 0.38 |
|  |  | (8.2-22.2) | (0.28-1.05) | (0.27-1.02) | | (4.0-12.3) | (0.87-6.27) | (0.91-6.56) | | (3.3-12.7) | (0.12-2.74) | (0.05-2.96) |
| North-East | 90 | 28.5 | 1.34 | 1.26 | | 4.7 | 0.43 | 0.32 | | 7.7 | 0.52 | 0.32 |
|  |  | (18.7-40.8) | (0.71-2.54) | (0.68-2.33) | | (2.0-11.0) | (0.11-1.65) | (0.08-1.32) | | (3.0-18.4) | (0.06-4.63) | (0.03-3.31) |
| **Place of residence** |  |  |  |  | |  |  |  | |  |  |  |
| Rural | 940 | 22.3 | 1 | 1 | | 10.2 | 1 | 1 | | 12.4 | 1 | 1 |
|  |  | (18.6-26.4) |  |  |  | (7.5-13.7) |  |  |  | (9.4-16.1) |  |  |
| Urban | 702 | 23.1 | 1.05 | 0.65 | | 11.8 | 1.25 | 1.39 | | 7.6 | 0.27 | 0.22 |
|  |  | (18.8-28.0) | (0.74-1.48) | (0.43-1.00) | | (9.1-15.2) | (0.54-1.31) | (0.68-2.84) | | (5.2-10.8) | (0.10-0.71) | (0.07-0.66) |
| *Primary: illiterate and <6th standard; Secondary: 6th to 10th standard; Higher secondary and above: 11th standard and above | | | | | | | | | | | | |
| **Homemaker/ retired/unemployed | | | | | | | | | | | | |
| ***professionals /managers/executives /self-employed | | | | | | | | | | | | |

| **Supplementary Table 2b**: **Prevalence (%) and determinants (aOR with 95% CI) of awareness, treatment, and control among those with hypertension for females** | | | | | | | | | | | | | |
| --- | --- | --- | --- | --- | --- | --- | --- | --- | --- | --- | --- | --- | --- |
| **Subgroups** | **Awareness** | | | | **Treatment** | | | | **Control** | | | | |
|  | **n** | **Prevalence (%)** | **UOR** | **AOR** | | **Prevalence (%)** | **UOR** | **AOR** | | **Prevalence (%)** | **UOR** | **AOR** |  |
|  |  | **(95% CI)** | **(95% CI)** | **(95% CI)** | | **(95% CI)** | **(95% CI)** | **(95% CI)** | | **(95% CI)** | **(95% CI)** | **(95% CI)** |  |
| **Total** | 1375 | 34.1 |  |  | | 18.8 |  |  | | 15.3 |  |  |  |
|  |  | (30.7 - 37.7) |  |  |  | (16.2 - 21.7) |  |  |  | (13.0 - 17.8) |  |  |  |
| **Age groups (in years)** |  |  |  |  | |  |  |  | |  |  |  |  |
| 18-29 | 158 | 27.9 | 1 | 1 | | 5.3 | 1 | 1 | | 21.8 | 1 | 1 |  |
|  |  | (19.4-38.3) |  |  |  | (1.7-15.5) |  |  |  | (14.3-31.8) |  |  |  |
| 30-49 | 600 | 27.4 | 0.97 | 0.98 | | 11 | 2.85 | 2.87 | | 12.5 | 0.58 | 1.41 |  |
|  |  | (22.7-32.6) | (0.58-1.64) | (0.57-1.67) | | (8.0-14.8) | (0.78-10.49) | (0.65-12.68) | | (9.6-16.2) | (0.05-6.43) | (0.11-17.47) |  |
| 50-69 | 617 | 42.3 | **1.89** | **1.89** | | 30 | **10.43** | **9.17** | | 16.3 | 0.39 | 0.88 |  |
|  |  | (37.3-47.4) | **(1.16-3.08)** | **(1.10-3.21)** | | (25.6-34.7) | **(2.87-37.90)** | **(2.10-39.94)** | | (12.9-20.3) | (0.04-3.66) | (0.09-8.93) |  |
| **Education*** |  |  |  |  | |  |  |  | |  |  |  |  |
| Primary | 923 | 31.8 | 1 | 1 | | 18.3 | 1 | 1 | | 13.4 | 1 | 1 |  |
|  |  | (28.1-35.7) |  |  |  | (15.3-21.7) |  |  |  | (11.0-16.2) |  |  |  |
| Secondary | 307 | 36.8 | 1.25 | 0.95 | | 18.8 | 0.77 | 0.75 | | 17.3 | 0.97 | 1.15 |  |
|  |  | (29.9-44.3) | (0.89-1.76) | (0.64-1.42) | | (14.2-24.5) | (0.44-1.32) | (0.40-1.41) | | (12.6-23.3) | (0.49-1.95) | (0.55-2.40) |  |
| Higher secondary and above | 144 | 43.8 | **1.67** | 1.11 | | 22.5 | 0.77 | 0.92 | | 22.9 | 0.83 | 1.17 |  |
|  |  | (34.3-53.7) | **(1.10-2.53)** | (0.68-1.81) | | (15.0-32.2) | (0.38-1.56) | (0.35-2.39) | | (16.1-31.5) | (0.32-2.15) | (0.40-3.41) |  |
| **Occupation** |  |  |  |  | |  |  |  | |  |  |  |  |
| Skilled/unskilled labourers | 274 | 24.3 | 1 | 1 | | 12.9 | 1 | 1 | | 10.3 | 1 | 1 |  |
|  |  | (17.9-32.1) |  |  |  | (8.4-19.3) |  |  |  | (6.2-16.7) |  |  |  |
| Not working** | 1002 | 36.9 | **1.82** | 1.27 | | 20.4 | 1.10 | 0.95 | | 16.8 | 0.77 | 1.12 |  |
|  |  | (33.0-40.9) | **(1.19-2.77)** | (0.79-2.02) | | (17.4-23.8) | (0.52-2.31) | (0.36-2.50) | | (14.2-19.9) | (0.31-1.92) | (0.46-2.72) |  |
| Others*** | 99 | 33.1 | 1.54 | 0.97 | | 19.1 | 1.20 | 1.12 | | 13.2 | 0.44 | 0.44 |  |
|  |  | (22.6-45.6) | (0.79-3.00) | (0.48-1.97) | | (10.7-31.7) | (0.38-3.85) | (0.30-4.15) | | (7.3-22.5) | (0.09-2.09) | (0.10-2.00) |  |
| **Wealth index Quintiles** |  |  |  |  | |  |  |  | |  |  |  |  |
| Q1 | 384 | 20.3 | 1 | 1 | | 7.8 | 1 | 1 | | 10.6 | 1 | 1 |  |
|  |  | (15.4-26.2) |  |  |  | (4.7-12.9) |  |  |  | (7.1-15.6) |  |  |  |
| Q2 | 292 | 31.5 | **1.81** | **1.87** | | 17.2 | 1.90 | 1.41 | | 13.8 | 0.54 | 0.63 |  |
|  |  | (25.2-38.7) | **(1.14-2.88)** | **(1.16-3.02)** | | (12.5-23.2) | (0.84-4.32) | (0.52-3.81) | | (9.2-20.2) | (0.15-1.93) | (0.18-2.14) |  |
| Q3 | 240 | 30.1 | **1.69** | **1.68** | | 18.7 | **2.59** | 1.94 | | 13.8 | 0.54 | 0.62 |  |
|  |  | (24.0-37.1) | **(1.06-2.70)** | **(1.02-2.77)** | | (14.2-24.1) | **(1.06-6.31)** | (0.71-5.31) | | (9.6-19.5) | (0.15-1.88) | (0.17-2.28) |  |
| Q4 | 232 | 46.6 | **3.43** | **3.23** | | 26.9 | 2.16 | 2.41 | | 18.5 | 0.57 | 0.64 |  |
|  |  | (39.0-54.4) | **(2.16-5.46)** | **(1.96-5.33)** | | (20.8-34.0) | (0.99-4.73) | (0.92-6.28) | | (13.8-24.3) | (0.18-1.75) | (0.19-2.17) |  |
| Q5 | 226 | 52.4 | **4.33** | **3.50** | | 31.5 | **2.40** | 2.30 | | 23.4 | 0.46 | 0.52 |  |
|  |  | (45.6-59.1) | **2.79-6.70)** | **(2.03-6.02)** | | (25.0-38.8) | **(1.06-5.43)** | (0.83-6.41) | | (17.8-30.1) | (0.14-1.53) | (0.14-1.90) |  |
| **Regions of India** |  |  |  |  | |  |  |  | |  |  |  |  |
| Central | 276 | 29.6 | 1 | 1 | | 10.1 | 1 | 1 | | 11.0 | 1 | 1 |  |
|  |  | (22.7-37.6) |  |  |  | (6.6-15.1) |  |  |  | (7.5-15.9) |  |  |  |
| East | 320 | 25.8 | 0.83 | 0.94 | | 11.9 | 1.65 | 1.78 | | 12.0 | **4.45** | **4.26** |  |
|  |  | (19.9-32.7) | (0.50-1.35) | (0.58-1.53) | | (8.1-17.0) | (0.78-3.51) | (0.78-4.04) | | (8.7-16.3) | **(1.47-13.50)** | **(1.35-13.47)** |  |
| North | 208 | 47.1 | **2.12** | 1.48 | | 26.9 | 2.58 | 2.08 | | 25.2 | **4.09** | **4.33** |  |
|  |  | (40.0-54.3) | **(1.33-3.36)** | (0.91-2.42) | | (20.5-34.4) | **(1.16-5.71)** | (0.83-5.18) | | (19.1-32.3) | **(1.41-11.87)** | **(1.39-13.47)** |  |
| South | 408 | 36.8 | 1.38 | 1.20 | | 25.4 | **4.32** | **3.15** | | 15.5 | 2.34 | **2.85** |  |
|  |  | (30.3-43.7) | (0.87-2.19) | (0.73-1.96) | | (20.2-31.4) | **(1.95-9.57)** | **(1.47-6.75)** | | (11.4-20.8) | (0.95-5.75) | **(1.06-7.67)** |  |
| West | 112 | 26.3 | 0.85 | 0.69 | | 17.8 | **4.06** | **3.85** | | 10.0 | 1.92 | 2.09 |  |
|  |  | (19.3-34.7) | (0.50-1.45) | (0.41-1.16) | | (11.7-26.1) | **(1.41-11.69)** | **(1.40-10.58)** | | (5.9-16.3) | (0.65-5.62) | (0.67-6.54) |  |
| North-East | 52 | 53.7 | **2.75** | **2.58** | | 26.6 | 1.90 | 2.11 | | 27.8 | 1.54 | 1.73 |  |
|  |  | (32.2-73.8) | **(1.05-7.20)** | **(1.05-6.33)** | | (12.5-48.0) | (0.64-5.67) | (0.62-7.14) | | (12.8-50.2) | (0.23-10.26) | (0.24-12.42) |  |
| **Place of residence** |  |  |  |  | |  |  |  | |  |  |  |  |
| Rural | 873 | 30.1 | 1 | 1 | | 15.4 | 1 | 1 | | 14.4 | 1 | 1 |  |
|  |  | (26.1-34.5) |  |  |  | (12.3-19.1) |  |  |  | (11.6-17.7) |  |  |  |
| Urban | 502 | 41.1 | 1.62 | 1.06 | | 24.9 | 1.47 | 1.09 | | 16.8 | 0.70 | 0.83 |  |
|  |  | (35.4-47.0) | (1.18-2.21) | (0.77-1.46) | | (20.4-29.9) | (0.87-2.47) | (0.59-2.01) | | (13.3-21.1) | (0.37-1.32) | (0.39-1.74) |  |
| *Primary: illiterate and <6th standard; Secondary: 6th to 10th standard; Higher secondary and above: 11th standard and above | | | | | | | | | | | | | |
| **Homemaker/ retired/unemployed | | | | | | | | | | | | | |
| ***professionals /managers/executives /self-employed | | | | | | | | | | | | | |

| **Supplementary Table 2c**: **Prevalence (%) and determinants (aOR with 95% CI) of awareness, treatment, and control among those with hypertension in urban areas** | | | | | | | | | | | | |
| --- | --- | --- | --- | --- | --- | --- | --- | --- | --- | --- | --- | --- |
| **Subgroups** | **Awareness** | | | | **Treatment** | | | | **Control** | | | |
|  | **n** | **Prevalence (%)** | **UOR** | **AOR** | | **Prevalence (%)** | **UOR** | **AOR** | | **Prevalence (%)** | **UOR** | **AOR** |
|  |  | **(95% CI)** | **(95% CI)** | **(95% CI)** | | **(95% CI)** | **(95% CI)** | **(95% CI)** | | **(95% CI)** | **(95% CI)** | **(95% CI)** |
| **Total** | 1204 | 30.6 |  |  | | 17.3 |  |  | | 11.4 |  |  |
|  |  | (26.6 - 34.9) |  |  |  | (14.7 - 20.2) |  |  |  | (9.2 - 14.1) |  |  |
| **Age groups (in years)** |  |  |  |  | |  |  |  | |  |  |  |
| 18-29 | 135 | 25.6 | 1 | 1 | | 3.2 | 1 | 1 | | 14.2 | 1 | 1 |
|  |  | (16.4-37.6) |  |  |  | (0.6-15.2) |  |  |  | (7.7-24.7) |  |  |
| 30-49 | 597 | 24.1 | 0.92 | 0.86 | | 10.2 | 5.25 | 8.93 | | 8.4 | 1.45 | 1.97 |
|  |  | (19.3-29.6) | (0.49-1.73) | (0.46-1.58) | | (7.5-13.8) | (0.85-32.43) | (1.08-73.92) | | (5.9-11.9) | (0.69-3.04) | (0.94-4.12) |
| 50-69 | 472 | 40.2 | 1.95 | 1.64 | | 30.2 | 21.30 | 29.21 | | 14.4 | 0.0 | 0.0 |
|  |  | (33.4-47.4) | (1.05-3.65) | (0.88-3.03) | | (24.7-36.3) | (3.47-130.76) | (3.63-235.26) | | (10.7-19.0) | (0.0-0.0) | (0.0-0.0) |
| **Sex** |  |  |  |  | |  |  |  | |  |  |  |
| Male | 702 | 23.1 | 1 | 1 | | 11.8 | 1 | 1 | | 7.6 | 1 | 1 |
|  |  | (18.8-28.0) |  |  |  | (9.1-15.2) |  |  |  | (5.2-10.8) |  |  |
| Female | 502 | 41.1 | **2.32** | **1.94** | | 24.9 | 1.46 | 0.91 | | 16.8 | 1.06 | 0.58 |
|  |  | (35.4-47.0) | **(1.70-3.17)** | **(1.19-3.14)** | | (20.4-29.9) | (0.80-2.65) | (0.41-2.05) | | (13.3-21.1) | (0.45-2.49) | (0.25-1.35) |
| **Education*** |  |  |  |  | |  |  |  | |  |  |  |
| Primary | 395 | 32.0 | 1 | 1 | | 20 | 1 | 1 | | 14.4 | 1 | 1 |
|  |  | (26.5-38.0) |  |  |  | (15.9-24.9) |  |  |  | (10.5-19.5) |  |  |
| Secondary | 402 | 27.7 | 0.82 | 0.80 | | 16.7 | 0.91 | 1.29 | | 8.8 | 0.75 | 0.67 |
|  |  | (21.9-34.4) | (0.59-1.13) | (0.51-1.24) | | (12.4-22.1) | (0.42-1.96) | (0.58-2.87) | | (6.3-12.2) | (0.34-1.66) | (0.29-1.55) |
| Higher secondary and above | 405 | 32.2 | 1.01 | 0.97 | | 15.2 | 0.53 | 1.34 | | 11.1 | 0.74 | 0.68 |
|  |  | (26.9-37.9) | (0.73-1.39) | (0.65-1.44) | | (11.1-20.3) | (0.25-1.12) | (0.47-3.82) | | (7.4-16.4) | (0.30-1.81) | (0.27-1.72) |
| **Occupation** |  |  |  |  | |  |  |  | |  |  |  |
| Skilled/unskilled labourers | 309 | 16.5 | 1 | 1 | | 7.2 | 1 | 1 | | 7.0 | 1 | 1 |
|  |  | (11.4-23.2) |  |  |  | (4.1-12.3) |  |  |  | (4.1-11.8) |  |  |
| Not working** | 478 | 42.2 | **3.70** | **2.00** | | 27.1 | 2.32 | 3.02 | | 17.9 | 1.10 | 1.73 |
|  |  | (36.5-48.0) | **(2.29-5.97)** | **(1.13-3.55)** | | (22.5-32.3) | (0.92-5.85) | (0.91-10.09) | | (14.1-22.5) | (0.40-2.99) | (0.67-4.47) |
| Others*** | 415 | 27.8 | **1.95** | 1.62 | | 13.4 | 1.20 | 1.69 | | 7.2 | 0.72 | 0.59 |
|  |  | (21.5-35.1) | **(1.16-3.29)** | (0.94-2.78) | | (9.2-19.2) | (0.42-3.42) | (0.46-6.24) | | (4.6-11.3) | (0.20-2.65) | (0.17-2.03) |
| **Wealth index Quintiles** |  |  |  |  | |  |  |  | |  |  |  |
| Q1 | 117 | 16.7 | 1 | 1 | | 14.1 | 1 | 1 | | 4.3 | 1 | 1 |
|  |  | (9.2-28.3) |  |  |  | (7.2-25.7) |  |  |  | (1.4-12.8) |  |  |
| Q2 | 169 | 27 | 1.84 | 2.19 | | 15.3 | 0.24 | 0.39 | | 12.2 | 2.98 | 4.54 |
|  |  | (19.3-36.3) | (0.87-3.89) | (0.87-5.48) | | (9.7-23.5) | (0.06-0.96) | (0.08-1.96) | | (6.6-21.5) | (0.38-23.24) | (0.48-42.58) |
| Q3 | 184 | 22.5 | 1.45 | 1.72 | | 11.2 | 0.18 | 0.21 | | 7.9 | 2.08 | 2.51 |
|  |  | (15.5-31.6) | (0.60-3.50) | (0.58-5.11) | | (7.8-15.8) | (0.05-0.73) | (0.05-0.80) | | (4.9-12.5) | (0.28-15.42) | (0.26-23.88) |
| Q4 | 308 | 32.3 | **2.38** | **2.74** | | 18.4 | 0.24 | 0.44 | | 12.8 | 2.69 | 3.31 |
|  |  | (26.4-38.8) | **(1.15-4.90)** | **(1.12-6.70)** | | (14.0-23.7) | (0.06-0.91) | (0.12-1.59) | | (8.6-18.7) | (0.38-19.22) | (0.38-28.57) |
| Q5 | 426 | 38.1 | **3.07** | **3.29** | | 20.7 | 0.22 | 0.33 | | 13.6 | 2.82 | 4.49 |
|  |  | (32.4-44.1) | **(1.47-6.38)** | **(1.27-8.54)** | | (16.1-26.2) | (0.06-0.82) | (0.09-1.26) | | (10.2-17.9) | (0.41-19.45) | (0.53-37.76) |
| **Regions of India** |  |  |  |  | |  |  |  | |  |  |  |
| Central | 240 | 29.3 | 1 | 1 | | 11.8 | 1 | 1 | | 9.8 | 1 | 1 |
|  |  | (20.5-40.1) |  |  |  | (7.6-17.7) |  |  |  | (6.0-15.7) |  |  |
| East | 127 | 24.9 | 0.80 | 0.67 | | 13.4 | 1.74 | 1.27 | | 12.3 | 0.52 | 0.49 |
|  |  | (17.3-34.4) | (0.41-1.55) | (0.33-1.39) | | (8.3-20.9) | (0.72-4.23) | (0.42-3.89) | | (7.6-19.2) | (0.17-1.58) | (0.13-1.87) |
| North | 161 | 34.2 | 1.25 | 1.00 | | 19.2 | 1.91 | 1.34 | | 15.1 | 0.70 | 0.81 |
|  |  | (23.6-46.6) | (0.62-2.53) | (0.50-2.03) | | (12.0-29.3) | (0.88-4.17) | (0.54-3.33) | | (8.3-25.8) | (0.24-2.08) | (0.27-2.42) |
| South | 486 | 32.5 | 1.16 | 1.16 | | 21.1 | **2.76** | 2.47 | | 12.3 | 0.61 | 0.83 |
|  |  | (25.9-39.9) | (0.65-2.06) | (0.64-2.10) | | (16.9-26.1) | **(1.22-6.25)** | (0.97-6.27) | | (8.9-16.8) | (0.21-1.72) | (0.30-2.29) |
| West | 130 | 18.6 | 0.55 | 0.59 | | 12.2 | **2.84** | 3.53 | | 8.1 | 0.82 | 0.89 |
|  |  | (13.3-25.4) | (0.29-1.03) | (0.31-1.13) | | (7.2-19.9) | **(1.01-8.09)** | (1.31-9.49) | | (4.8-13.4) | (0.28-2.39) | (0.28-2.83) |
| North-East | 59 | 48.4 | **2.26** | **2.16** | | 21.5 | 1.19 | 0.89 | | 6.0 | 0.14 | 0.12 |
|  |  | (37.0-59.9) | **(1.16-4.40)** | **(1.03-4.57)** | | (12.1-35.3) | (0.49-2.89) | (0.24-3.30) | | (1.9-17.7) | (0.01-1.15) | (0.01-1.16) |
| *Primary: illiterate and <6th standard; Secondary: 6th to 10th standard; Higher secondary and above: 11th standard and above | | | | | | | | | | | | |
| **Homemaker/ retired/unemployed | | | | | | | | | | | | |
| ***professionals /managers/executives /self-employed | | | | | | | | | | | | |

| **Supplementary Table 2d**: **Prevalence (%) and determinants (aOR with 95% CI) of awareness, treatment, and control among those with hypertension in rural areas** | | | | | | | | | | |
| --- | --- | --- | --- | --- | --- | --- | --- | --- | --- | --- |
| **Subgroups** | **Awareness** | | | | **Treatment** | | | **Control** | | |
|  | **n** | **Prevalence (%)** | **UOR** | **AOR** | **Prevalence (%)** | **UOR** | **AOR** | **Prevalence (%)** | **UOR** | **AOR** |
|  |  | **(95% CI)** | **(95% CI)** | **(95% CI)** | **(95% CI)** | **(95% CI)** | **(95% CI)** | **(95% CI)** | **(95% CI)** | **(95% CI)** |
| **Total** | 1813 | 26.0 |  |  | 12.7 |  |  | 13.3 |  |  |
|  |  | (23.2 - 29.1) |  |  | (10.3 - 15.5) |  |  | (11.2 - 15.7) |  |  |
| **Age groups (in years)** |  |  |  |  |  |  |  |  |  |  |
| 18-29 | 277 | 16.2 | 1 | 1 | 6.4 | 1 | 1 | 14 | 1 | 1 |
|  |  | (10.9-23.4) |  |  | (2.7-14.5) |  |  | (9.0-21.4) |  |  |
| 30-49 | 835 | 21.1 | 1.39 | 1.54 | 8.8 | 1.10 | 0.74 | 12.4 | 0.37 | 0.61 |
|  |  | (17.3-25.6) | (0.78-2.46) | (0.85-2.79) | (6.1-12.6) | (0.36-3.34) | (0.24-2.28) | (9.2-16.6) | (0.05-2.67) | (0.07-5.04) |
| 50-69 | 701 | 35.8 | **2.89** | **3.17** | 19.8 | 1.90 | 1.39 | 14.1 | 0.17 | 0.36 |
|  |  | (31.5-40.4) | **(1.77-4.71)** | **(1.78-5.66)** | (15.8-24.5) | (0.62-5.82) | (0.43-4.52) | (11.3-17.5) | (0.03-1.11) | (0.04-3.08) |
| **Sex** |  |  |  |  |  |  |  |  |  |  |
| Male | 940 | 22.3 | 1 | 1 | 10.2 | 1 | 1 | 12.4 | 1 | 1 |
|  |  | (18.6-26.4) |  |  | (7.5-13.7) |  |  | (9.4-16.1) |  |  |
| Female | 873 | 30.1 | **1.51** | **1.50** | 15.4 | 1.24 | 2.06 | 14.4 | 0.41 | 0.53 |
|  |  | (26.1-34.5) | **(1.12-2.03)** | **(1.01-2.21)** | (12.3-19.1) | (0 .78-1.97) | (0.97-4.37) | (11.6-17.7) | (0.18-0.96) | (0.19-1.42) |
| **Education*** |  |  |  |  |  |  |  |  |  |  |
| Primary | 1089 | 25.8 | 1 | 1 | 12.3 | 1 | 1 | 12.5 | 1 | 1 |
|  |  | (22.7-29.3) |  |  | (9.8-15.3) |  |  | (10.2-15.3) |  |  |
| Secondary | 481 | 24.4 | 0.93 | 1.09 | 12.5 | 1.16 | 1.03 | 12.3 | 1.13 | 0.91 |
|  |  | (18.8-31.0) | (0.64-1.34) | (0.72-1.66) | (8.5-18.0) | (0.66-2.02) | (0.55-1.94) | (8.7-17.2) | (0.51-2.52) | (0.36-2.33) |
| Higher secondary and above | 240 | 29.9 | 1.23 | 1.40 | 15.0 | 1.10 | 1.24 | 19.2 | 3.47 | 1.86 |
|  |  | (21.7-39.7) | (0.78-1.92) | (0.74-2.65) | (9.1-23.6) | (0.53-2.28) | (0.49-3.15) | (12.1-29.1) | (0.97-12.38) | (0.33-10.51) |
| **Occupation** |  |  |  |  |  |  |  |  |  |  |
| Skilled/unskilled labourers | 665 | 17.4 | 1 | 1 | 7.8 | 1 | 1 | 9.2 | 1 | 1 |
|  |  | (13.9-21.5) |  |  | (5.5-11.1) |  |  | (6.7-12.5) |  |  |
| Not working** | 760 | 32.3 | **2.27** | 1.45 | 15.5 | 1.12 | 0.79 | 15.2 | 0.84 | 1.35 |
|  |  | (27.9-37.1) | **(1.61-3.20)** | (0.94-2.24) | (12.1-19.5) | (0.63-1.99) | (0.38-1.65) | (12.1-19.1) | (0.37-1.92) | (0.50-3.64) |
| Others*** | 388 | 28.7 | 1.91 | 1.38 | 15.5 | 1.43 | 1.71 | 16.6 | 1.78 | 1.78 |
|  |  | (21.9-36.6) | (1.23-2.97) | (0.87-2.19) | (9.7-23.9) | (0.67-3.06) | (0.68-4.29) | (10.8-24.7) | (0.47-6.72) | (0.44-7.25) |
| **Wealth index Quintiles** |  |  |  |  |  |  |  |  |  |  |
| Q1 | 606 | 17.4 | 1 | 1 | 5.2 | 1 | 1 | 10.4 | 1 | 1 |
|  |  | (13.8-21.7) |  |  | (3.2-8.5) |  |  | (7.6-14.1) |  |  |
| Q2 | 459 | 25.9 | **1.66** | **1.74** | 13.1 | **2.36** | **2.36** | 12.1 | 0.52 | 0.33 |
|  |  | (20.8-31.7) | **(1.10-2.51)** | **(1.14-2.65)** | (8.8-19.0) | **(1.11-5.03)** | **(1.06-5.24)** | (7.9-18.0) | (0.14-1.91) | (0.08-1.38) |
| Q3 | 346 | 24.0 | **1.50** | 1.50 | 13.5 | **3.01** | **2.97** | 13.0 | 0.60 | 0.49 |
|  |  | (18.8-30.0) | **(1.00-2.25)** | (0.98-2.29) | (9.9-18.3) | **(1.32-6.87)** | **(1.28-6.89)** | (9.4-17.8) | (0.17-2.05) | (0.10-2.42) |
| Q4 | 264 | 34.7 | **2.52** | **2.20** | 19.2 | **2.87** | **2.71** | 16.0 | 0.59 | 0.34 |
|  |  | (27.8-42.2) | **(1.63-3.90)** | **(1.37-3.52)** | (13.6-26.4) | **(1.42-5.82)** | **(1.27-5.78)** | (10.3-23.9) | (0.19-1.87) | (0.08-1.48) |
| Q5 | 138 | 53.5 | **5.47** | **4.22** | 29.6 | **2.87** | **3.03** | 25.9 | 0.48 | 0.28 |
|  |  | (41.5-65.1) | **(3.05-9.81)** | **(2.18-8.18)** | (17.8-44.9) | **(1.08-7.61)** | **(1.03-8.86)** | (17.0-37.3) | (0.13-1.78) | (0.06-1.30) |
| **Regions of India** |  |  |  |  |  |  |  |  |  |  |
| Central | 373 | 23.7 | 1 | 1 | 6.4 | 1 | 1 | 13.2 | 1 | 1 |
|  |  | (18.7-29.7) |  |  | (4.0-9.9) |  |  | (9.3-18.4) |  |  |
| East | 507 | 22.2 | 0.92 | 1.00 | 11.8 | **3.08** | **3.56** | 12.8 | 3.91 | 3.53 |
|  |  | (17.6-27.6) | (0.60-1.40) | (0.65-1.54) | (8.1-16.9) | **(1.46-6.50)** | **(1.63-7.79)** | (8.8-18.2) | (0.83-18.17) | (0.75-16.65) |
| North | 307 | 36.3 | 1.83 | 1.26 | 18.9 | **2.96** | 2.55 | 18.2 | 2.39 | 2.94 |
|  |  | (29.7-43.6) | (1.19-2.82) | (0.82-1.94) | (12.8-27.2) | **(1.28-6.85)** | (0.97-6.67) | (13.5-24.0) | (0.64-8.90) | (0.84-10.35) |
| South | 367 | 28.0 | 1.25 | 1.13 | 17.7 | **4.71** | **3.75** | 11.1 | 1.29 | 1.96 |
|  |  | (21.2-35.9) | (0.77-2.01) | (0.72-1.78) | (11.5-26.3) | **(2.09-10.57)** | **(1.70-8.29)** | (7.5-16.1) | (0.38-4.38) | (0.59-6.48) |
| West | 176 | 18.2 | 0.72 | 0.62 | 10.2 | **3.47** | **4.15** | 7.6 | 1.22 | 1.51 |
|  |  | (12.0-26.7) | (0.40-1.28) | (0.35-1.09) | (6.3-16.2) | **(1.23-9.81)** | **(1.39-12.39)** | (4.0-13.9) | (0.28-5.22) | (0.32-7.12) |
| North-East | 82 | 29.9 | 1.37 | 1.38 | 6.3 | 0.73 | 0.70 | 21.4 | 7.14 | 13.51 |
|  |  | (18.1-45.2) | (0.66-2.84) | (0.75-2.55) | (2.5-14.9) | (0.27-2.01) | (0.27-1.81) | (10.0-40.2) | (0.87-58.72) | (1.51-120.73) |
| *Primary: illiterate and <6th standard; Secondary: 6th to 10th standard; Higher secondary and above: 11th standard and above | | | | | | | | | | |
| **Homemaker/ retired/unemployed | | | | | | | | | | |
| ***professionals /managers/executives /self-employed | | | | | | | | | | |

| **Supplementary Table 4a**: **Determinants of control of Hypertension (for those who are on treatment) among male population of India** | | | | | |
| --- | --- | --- | --- | --- | --- |
|  | **n** | **Not controlled %** | **Controlled %** | **Crude OR** | **Adjusted OR** |
|  |  | **(95% CI)** | **(95% CI)** | **(95% CI)** | **(95% CI)** |
| **Age group (in years)** |  |  |  |  |  |
| 30-49 | 69 | 40.1 | 59.9 | 1 | 1 |
|  |  | (23.1-59.8) | (40.2-76.9) |  |  |
| 18-29 | 14 | 30.1 | 69.9 | 1.56 | 1.01 |
|  |  | (4.3-80.4) | (19.6-95.7) | (0.14-17.04) | (0.15-6.76) |
| 50-69 | 96 | 58.5 | 41.5 | 0.48 | 0.41 |
|  |  | (43.8-71.8) | (28.2-56.2) | (0.18-1.27) | (0.13-1.28) |
| **Place of residence** |  |  |  |  |  |
| Urban | 83 | 66.1 | 33.9 | 1 | 1 |
|  |  | (50.6-78.8) | (21.2-49.4) |  |  |
| Rural | 96 | 34.6 | 65.4 | **3.68** | **4.35** |
|  |  | (20.6-51.8) | (48.2-79.4) | **(1.41-9.56)** | **(1.58-12.00)** |
| **Body Mass Index** |  |  |  |  |  |
| Normal | 77 | 54.3 | 45.7 | 1 | 1 |
|  |  | (35.7-71.7) | (28.3-64.3) |  |  |
| Underweight | 6 | 20.4 | 79.6 | 4.63 | **10.85** |
|  |  | (3.1-67.5) | (32.5-96.9) | (0.51-42.08) | **(1.06-111.04)** |
| Overweight | 79 | 47.4 | 52.6 | 1.32 | 1.42 |
|  |  | (29.2-66.3) | (33.7-70.8) | (0.45-3.85) | (0.50-3.99) |
| Obese | 14 | 34.9 | 65.1 | 2.21 | 9.65 |
|  |  | (15.9-60.3) | (39.7-84.1) | (0.61-7.96) | (0.90-103.79) |
| **Waist circumference** |  |  |  |  |  |
| Not Raised | 83 | 48.5 | 51.5 | 1 | 1 |
|  |  | (30.9-66.4) | (33.6-69.1) |  |  |
| Raised | 95 | 49.8 | 50.2 | 0.95 | 0.95 |
|  |  | (35.2-64.5) | (35.5-64.8) | (0.38-2.37) | (0.31-2.93) |
| **Physical Activity** |  |  |  |  |  |
| Adequate | 85 | 46.4 | 53.6 | 1 | 1 |
|  |  | (29.2-64.5) | (35.5-70.8) |  |  |
| Inadequate | 94 | 51.8 | 48.2 | 0.81 | 2.85 |
|  |  | (36.7-66.6) | (33.4-63.3) | (0.32-2.07) | (0.96-8.48) |
| **Current Smoked Tobacco use** |  |  |  |  |  |
| Non-current | 139 | 53.8 | 46.2 | 1 | 1 |
|  |  | (40.8-66.3) | (33.7-59.2) |  |  |
| Current | 40 | 33.5 | 66.5 | 2.31 | 1.52 |
|  |  | (14.4-60.1) | (39.9-85.6) | (0.70-7.56) | (0.41-5.71) |
| **Current Smokeless Tobacco use** |  |  |  |  |  |
| Non-current | 149 | 46.2 | 53.8 | 1 | 1 |
|  |  | (33.3-59.7) | (40.3-66.7) |  |  |
| Current | 30 | 64.0 | 36.0 | 0.48 | 0.25 |
|  |  | (41.3-81.8) | (18.2-58.7) | (0.17-1.37) | (0.07-0.83) |
| **Hazardous drinking** |  |  |  |  |  |
| No | 164 | 51.0 | 49.0 | 1 | 1 |
|  |  | (38.3-63.5) | (36.5-61.7) |  |  |
| Yes | 15 | 30.2 | 69.8 | 2.40 | 1.26 |
|  |  | (7.7-69.1) | (30.9-92.3) | (0.43-13.36) | (0.19-8.33) |
| **Dietary salt related practices** |  |  |  |  |  |
| At least one* measure taken on regularly | 118 | 54.3 | 45.7 | 1 | 1 |
|  |  | (38.1-69.7) | (30.3-61.9) |  |  |
| None | 61 | 39.4 | 60.6 | 1.82 | **5.81** |
|  |  | (24.8-56.2) | (43.8-75.2) | (0.71-4.67) | **(1.98-17.01)** |
| **Co-morbidities** |  |  |  |  |  |
| No other self-reported chronic disease | 98 | 36.6 | 63.4 | 1 | 1 |
|  |  | (22.6-53.4) | (46.6-77.4) |  |  |
| Yes | 81 | 64.6 | 35.4 | 0.32 | 0.60 |
|  |  | (48.5-77.9) | (22.1-51.5) | (0.12-0.81) | (0.24-1.51) |
| **Adherence***** |  |  |  |  |  |
| ≥11 days | 121 | 58.6 | 41.4 | 1 | 1 |
|  |  | (44.8-71.2) | (28.8-55.2) |  |  |
| 6 to 10 days | 39 | 33.3 | 66.7 | 2.83 | 1.96 |
|  |  | (14.3-60.0) | (40.0-85.7) | (0.82-9.68) | (0.50-7.73) |
| ≤5 days | 19 | 22.4 | 77.6 | 4.89 | 4.66 |
|  |  | (6.3-55.3) | (44.7-93.7) | (1.06-22.49) | (0.91-23.77) |
| **Physicians consulted for Hypertension** |  |  |  |  |  |
| Govt. health facility | 27 | 46.6 | 53.4 | 1 | 1 |
|  |  | (25.8-68.7) | (31.3-74.2) |  |  |
| PVT/NGO health facility | 136 | 48.6 | 51.4 | 0.93 | 1.41 |
|  |  | (34.0-63.4) | (36.6-66.0) | (0.31-2.80) | (0.30-6.63) |
| No | 16 | 59.3 | 40.7 | 0.60 | 0.90 |
|  |  | (31.7-82.0) | (18.0-68.3) | (0.14-2.59) | (0.12-7.07) |
| **Source of medicines** |  |  |  |  |  |
| Govt. facility | 22 | 32.3 | 67.7 | 1 | 1 |
|  |  | (15.6-55.2) | (44.8-84.4) |  |  |
| Chemist/ Private/ NGO Dispensary | 150 | 49.9 | 50.1 | 0.48 | 0.40 |
|  |  | (36.4-63.3) | (36.7-63.6) | (0.16-1.42) | (0.08-1.88) |
| *Measures to control salt intake: Limit consumption of high salt containing food/Look at the salt or sodium content on food labels/use of low salt or sodium alternatives/Use spices other than salt/avoid foods prepared outside home/other measures. | | | | | |
| **Diabetes/ hypercholesterolemia / CVD | | | | | |
| ***adherence to treatment in last 2 weeks | | | | | |

| **Supplementary Table 4b**: **Determinants of control of Hypertension (for those who are on treatment) among female population of India** | | | | | |
| --- | --- | --- | --- | --- | --- |
|  | **n** | **Not controlled %** | **Controlled %** | **Crude OR** | **Adjusted OR** |
|  |  | **(95% CI)** | **(95% CI)** | **(95% CI)** | **(95% CI)** |
| **Age group (in years)** |  |  |  |  |  |
| 30-49 | 66 | 55.8 | 44.2 | 1 | 1 |
|  |  | (40.2-70.3) | (29.7-59.8) |  |  |
| 18-29 | 8 | 40.0 | 60.0 | 1.73 | 1.71 |
|  |  | (6.6-86.3) | (13.7-93.4) | (0.16-19.16) | (0.14-21.42) |
| 50-69 | 185 | 63.6 | 36.4 | 0.67 | 0.53 |
|  |  | (54.2-72.0) | (28.0-45.8) | (0.33-1.34) | (0.25-1.17) |
| **Place of residence** |  |  |  |  |  |
| Urban | 125 | 65.9 | 34.1 | 1 | 1 |
|  |  | (55.1-75.3) | (24.7-44.9) |  |  |
| Rural | 134 | 56.1 | 43.9 | 1.44 | 1.24 |
|  |  | (44.6-67.0) | (33.0-55.4) | (0.76-2.73) | (0.61-2.51) |
| **Body Mass Index** |  |  |  |  |  |
| Normal | 83 | 62.8 | 37.2 | 1 | 1 |
|  |  | (49.2-74.6) | (25.4-50.8) |  |  |
| Underweight | 14 | 28.4 | 71.6 | **4.25** | **4.55** |
|  |  | (10.5-57.3) | (42.7-89.5) | **(1.14-15.87)** | **(1.03-20.07)** |
| Overweight | 107 | 63.3 | 36.7 | 0.98 | 1.23 |
|  |  | (50.4-74.4) | (25.6-49.6) | (0.48-2.01) | (0.58-2.63) |
| Obese | 49 | 59.8 | 40.2 | 1.13 | 1.24 |
|  |  | (44.5-73.4) | (26.6-55.5) | (0.48-2.68) | (0.45-3.38) |
| **Waist circumference** |  |  |  |  |  |
| Not Raised | 67 | 54.8 | 45.2 | 1 | 1 |
|  |  | (38.8-69.8) | (30.2-61.2) |  |  |
| Raised | 187 | 62.5 | 37.5 | 0.73 | 0.66 |
|  |  | (53.4-70.8) | (29.2-46.6) | (0.35-1.52) | (0.25-1.76) |
| **Physical Activity** |  |  |  |  |  |
| Adequate | 110 | 64.5 | 35.5 | 1 | 1 |
|  |  | (51.7-75.5) | (24.5-48.3) |  |  |
| Inadequate | 149 | 58.1 | 41.9 | 1.35 | 1.49 |
|  |  | (48.4-67.2) | (32.8-51.6) | (0.72-2.53) | (0.77-2.90) |
| **Current Smoked Tobacco use** |  |  |  |  |  |
| Non-current | 258 | 60.7 | 39.3 | 1 | 1 |
|  |  | (52.7-68.3) | (31.7-47.3) |  |  |
| Current | 1 | 100.0 | 0.0 | 0.0 | 0.0 |
|  |  | (100.0-100.0) | (0.0-0.0) | (0.0-0.0) | (0.0-0.0) |
| **Current Smokeless Tobacco use** |  |  |  |  |  |
| Non-current | 230 | 58.0 | 42.0 | 1 | 1 |
|  |  | (49.4-66.2) | (33.8-50.6) |  |  |
| Current | 29 | 83.2 | 16.8 | 0.28 | 0.24 |
|  |  | (63.9-93.3) | (6.7-36.1) | (0.09-0.81) | (0.09-0.68) |
| **Hazardous drinking** |  |  |  |  |  |
| No | 259 | 60.8 | 39.2 | 1 | 1 |
|  |  | (52.8-68.3) | (31.7-47.2) |  |  |
| Yes | 0 | 0.0 | 0.0 | 0.0 | 0.0 |
|  |  | (0.0-0.0) | (0.0-0.0) | (0.0-0.0) | (0.0-0.0) |
| **Dietary salt related practices** |  |  |  |  |  |
| At least one* measure taken on regularly | 145 | 59.1 | 40.9 | 1 | 1 |
|  |  | (49.3-68.2) | (31.8-50.7) |  |  |
| None | 114 | 63.0 | 37.0 | 0.89 | 0.80 |
|  |  | (51.0-73.6) | (26.4-49.0) | (0.50-1.59) | (0.43-1.49) |
| **Co-morbidities** |  |  |  |  |  |
| No other self-reported chronic disease | 166 | 64.6 | 35.4 | 1 | 1 |
|  |  | (54.0-73.9) | (26.1-46.0) |  |  |
| Yes | 93 | 54.1 | 45.9 | 1.50 | 2.08 |
|  |  | (41.7-66.1) | (33.9-58.3) | (0.76-2.96) | (0.90-4.83) |
| **Adherence***** |  |  |  |  |  |
| ≥11 days | 188 | 63.2 | 36.8 | 1 | 1 |
|  |  | (54.3-71.2) | (28.8-45.7) |  |  |
| 6 to 10 days | 42 | 56.5 | 43.5 | 1.28 | 1.00 |
|  |  | (34.5-76.3) | (23.7-65.5) | (0.49-3.31) | (0.36-2.78) |
| ≤5 days | 29 | 51.5 | 48.5 | 1.56 | 1.56 |
|  |  | (31.3-71.3) | (28.7-68.7) | (0.62-3.90) | (0.55-4.41) |
| **Physicians consulted for Hypertension** |  |  |  |  |  |
| Govt. health facility | 69 | 52.4 | 47.6 | 1 | 1 |
|  |  | (38.7-65.8) | (34.2-61.3) |  |  |
| PVT/NGO health facility | 166 | 63.7 | 36.3 | 0.63 | 0.41 |
|  |  | (54.2-72.4) | (27.6-45.8) | (0.33-1.18) | (0.15-1.15) |
| No | 24 | 64.8 | 35.2 | 0.62 | 0.30 |
|  |  | (39.0-84.2) | (15.8-61.0) | (0.19-2.00) | (0.09-1.01) |
| **Source of medicines** |  |  |  |  |  |
| Govt. facility | 57 | 56.3 | 43.7 | 1 | 1 |
|  |  | (41.2-70.2) | (29.8-58.8) |  |  |
| Chemist/ Private/ NGO Dispensary | 194 | 62.1 | 37.9 | 0.79 | 1.67 |
|  |  | (53.0-70.4) | (29.6-47.0) | (0.41-1.53) | (0.57-4.84) |
| *Measures to control salt intake: Limit consumption of high salt containing food/Look at the salt or sodium content on food labels/use of low salt or sodium alternatives/Use spices other than salt/avoid foods prepared outside home/other measures. | | | | | |
| **Diabetes/ hypercholesterolemia / CVD | | | | | |
| ***adherence to treatment in last 2 weeks | | | | | |

| **Supplementary Table 4c**: **Determinants of control of Hypertension (for those who are on treatment) among urban population of India** | | | | | |
| --- | --- | --- | --- | --- | --- |
|  | **n** | **Not controlled %** | **Controlled %** | **Crude OR** | **Adjusted OR** |
|  |  | **(95% CI)** | **(95% CI)** | **(95% CI)** | **(95% CI)** |
| **Age group (in years)** |  |  |  |  |  |
| 30-49 | 61 | 60.5 | 39.5 | 1 | 1 |
|  |  | (44.1-74.9) | (25.1-55.9) |  |  |
| 18-29 | 4 | 100.0 | 0.0 | 0.0 | 0.0 |
|  |  | (100.0-100.0) | (0.0-0.0) | (0.0-0.0) | (0.0-0.0) |
| 50-69 | 142 | 67.3 | 32.7 | 0.69 | 0.55 |
|  |  | (58.6-75.0) | (25.0-41.4) | (0.33-1.45) | (0.25-1.20) |
| **Sex** |  |  |  |  |  |
| Female | 125 | 65.9 | 34.1 | 1 | 1 |
|  |  | (55.1-75.3) | (24.7-44.9) |  |  |
| Male | 83 | 66.1 | 33.9 | 0.94 | 1.40 |
|  |  | (50.6-78.8) | (21.2-49.4) | (0.40-2.22) | (0.58-3.37) |
| **Body Mass Index** |  |  |  |  |  |
| Normal | 64 | 81.7 | 18.3 | 1 | 1 |
|  |  | (71.0-89.0) | (11.0-29.0) |  |  |
| Underweight | 3 | 29.4 | 70.6 | 10.71 | 22.74 |
|  |  | (11.4-57.3) | (42.7-88.6) | (2.84-40.32) | (3.80-135.96) |
| Overweight | 99 | 63.0 | 37.0 | 2.61 | 2.95 |
|  |  | (51.1-73.6) | (26.4-48.9) | (1.21-5.65) | (1.10-7.88) |
| Obese | 36 | 46.5 | 53.5 | 5.12 | 6.70 |
|  |  | (32.7-60.9) | (39.1-67.3) | (2.20-11.92) | (2.10-21.35) |
| **Waist circumference** |  |  |  |  |  |
| Not Raised | 55 | 75.0 | 25.0 | 1 | 1 |
|  |  | (59.8-85.8) | (14.2-40.2) |  |  |
| Raised | 147 | 62.2 | 37.8 | 1.82 | 0.92 |
|  |  | (53.1-70.5) | (29.5-46.9) | (0.80-4.13) | (0.29-2.95) |
| **Physical Activity** |  |  |  |  |  |
| Adequate | 73 | 66.4 | 33.6 | 1 | 1 |
|  |  | (52.4-78.0) | (22.0-47.6) |  |  |
| Inadequate | 135 | 65.8 | 34.2 | 1.05 | 1.55 |
|  |  | (57.0-73.6) | (26.4-43.0) | (0.54-2.05) | (0.81-2.95) |
| **Current Smoked Tobacco use** |  |  |  |  |  |
| Non-current | 202 | 65.8 | 34.2 | 1 | 1 |
|  |  | (57.8-73.0) | (27.0-42.2) |  |  |
| Current | 5 | 74.8 | 25.2 | 0.62 | 0.86 |
|  |  | (40.5-92.8) | (7.2-59.5) | (0.14-2.80) | (0.10-7.65) |
| **Current Smokeless Tobacco use** |  |  |  |  |  |
| Non-current | 187 | 64.2 | 35.8 | 1 | 1 |
|  |  | (55.7-71.9) | (28.1-44.3) |  |  |
| Current | 21 | 82.2 | 17.8 | 0.39 | 0.25 |
|  |  | (67.1-91.3) | (8.7-32.9) | (0.16-0.93) | (0.09-0.64) |
| **Hazardous drinking** |  |  |  |  |  |
| No | 205 | 65.9 | 34.1 | 1 | 1 |
|  |  | (58.0-73.1) | (26.9-42.0) |  |  |
| Yes | 3 | 70.3 | 29.7 | 0.79 | 1.74 |
|  |  | (22.1-95.2) | (4.8-77.9) | (0.09-6.80) | (0.04-75.19) |
| **Dietary salt related practices** |  |  |  |  |  |
| At least one* measure taken on regularly | 118 | 67.5 | 32.5 | 1 | 1 |
|  |  | (58.7-75.2) | (24.8-41.3) |  |  |
| None | 90 | 64.0 | 36.0 | 1.24 | 1.21 |
|  |  | (50.3-75.8) | (24.2-49.7) | (0.65-2.35) | (0.59-2.46) |
| **Co-morbidities** |  |  |  |  |  |
| No other self-reported chronic disease | 109 | 64.3 | 35.7 | 1 | 1 |
|  |  | (52.7-74.5) | (25.5-47.3) |  |  |
| Yes | 99 | 67.9 | 32.1 | 0.82 | 0.87 |
|  |  | (56.6-77.3) | (22.7-43.4) | (0.41-1.62) | (0.44-1.74) |
| **Adherence***** |  |  |  |  |  |
| ≥11 days | 173 | 66.3 | 33.7 | 1 | 1 |
|  |  | (57.5-74.0) | (26.0-42.5) |  |  |
| 6 to 10 days | 26 | 63.7 | 36.3 | 1.08 | 0.59 |
|  |  | (36.5-84.2) | (15.8-63.5) | (0.32-3.64) | (0.15-2.31) |
| ≤5 days | 9 | 67.9 | 32.1 | 0.89 | 0.28 |
|  |  | (42.3-86.0) | (14.0-57.7) | (0.30-2.66) | (0.07-1.14) |
| **Physicians consulted for Hypertension** |  |  |  |  |  |
| Govt. health facility | 45 | 59.1 | 40.9 | 1 | 1 |
|  |  | (42.4-73.9) | (26.1-57.6) |  |  |
| PVT/NGO health facility | 144 | 68.0 | 32.0 | 0.68 | 0.44 |
|  |  | (58.2-76.4) | (23.6-41.8) | (0.30-1.54) | (0.16-1.25) |
| No | 19 | 67.3 | 32.7 | 0.73 | 0.47 |
|  |  | (40.1-86.4) | (13.6-59.9) | (0.20-2.71) | (0.12-1.83) |
| **Source of medicines** |  |  |  |  |  |
| Govt. facility | 37 | 61.9 | 38.1 | 1 | 1 |
|  |  | (43.4-77.5) | (22.5-56.6) |  |  |
| Chemist/ Private/ NGO Dispensary | 164 | 66.2 | 33.8 | 0.83 | 1.31 |
|  |  | (56.8-74.4) | (25.6-43.2) | (0.34-2.02) | (0.40-4.24) |
| *Measures to control salt intake: Limit consumption of high salt containing food/Look at the salt or sodium content on food labels/use of low salt or sodium alternatives/Use spices other than salt/avoid foods prepared outside home/other measures. | | | | | |
| **Diabetes/ hypercholesterolemia / CVD | | | | | |
| ***adherence to treatment in last 2 weeks | | | | | |

**Supplementary Table 4d**: **Determinants of control of Hypertension (for those who are on treatment) among rural population of India**

|  | **n** | **Not controlled %** | **Controlled %** | **Crude OR** | **Adjusted OR** |
| --- | --- | --- | --- | --- | --- |
|  |  | **(95% CI)** | **(95% CI)** | **(95% CI)** | **(95% CI)** |
| **Age group (in years)** |  |  |  |  |  |
| 30-49 | 74 | 37.1 | 62.9 | 1 | 1 |
|  |  | (22.1-55.2) | (44.8-77.9) |  |  |
| 18-29 | 18 | 17.9 | 82.1 | 2.70 | 2.79 |
|  |  | (3.2-59.5) | (40.5-96.8) | (0.37-19.47) | (0.40-19.20) |
| 50-69 | 139 | 56.2 | 43.8 | 0.46 | 0.40 |
|  |  | (45.4-66.5) | (33.5-54.6) | (0.21-1.03) | (0.16-1.01) |
| **Sex** |  |  |  |  |  |
| Female | 134 | 56.1 | 43.9 | 1 | 1 |
|  |  | (44.6-67.0) | (33.0-55.4) |  |  |
| Male | 96 | 34.6 | 65.4 | 2.42 | 3.72 |
|  |  | (20.6-51.8) | (48.2-79.4) | (1.04-5.59) | (1.48-9.31) |
| **Body Mass Index** |  |  |  |  |  |
| Normal | 97 | 43.6 | 56.4 | 1 | 1 |
|  |  | (30.3-57.8) | (42.2-69.7) |  |  |
| Underweight | 17 | 25.4 | 74.6 | 2.27 | 2.93 |
|  |  | (9.2-53.4) | (46.6-90.8) | (0.58-8.86) | (0.65-13.15) |
| Overweight | 87 | 49.2 | 50.8 | 0.80 | 0.55 |
|  |  | (31.3-67.2) | (32.8-68.7) | (0.34-1.87) | (0.17-1.81) |
| Obese | 27 | 64.5 | 35.5 | 0.42 | 0.54 |
|  |  | (40.0-83.2) | (16.8-60.0) | (0.13-1.34) | (0.11-2.79) |
| **Waist circumference** |  |  |  |  |  |
| Not Raised | 95 | 37.6 | 62.4 | 1 | 1 |
|  |  | (24.2-53.2) | (46.8-75.8) |  |  |
| Raised | 135 | 53.9 | 46.1 | 0.52 | 0.74 |
|  |  | (42.5-64.9) | (35.1-57.5) | (0.26-1.04) | (0.22-2.48) |
| **Physical Activity** |  |  |  |  |  |
| Adequate | 122 | 50.8 | 49.2 | 1 | 1 |
|  |  | (37.5-64.0) | (36.0-62.5) |  |  |
| Inadequate | 108 | 43.0 | 57.0 | 1.37 | 2.28 |
|  |  | (30.6-56.4) | (43.6-69.4) | (0.68-2.75) | (1.03-5.07) |
| **Current Smoked Tobacco use** |  |  |  |  |  |
| Non-current | 194 | 50.6 | 49.4 | 1 | 1 |
|  |  | (40.5-60.6) | (39.4-59.5) |  |  |
| Current | 36 | 28.5 | 71.5 | 2.57 | 0.98 |
|  |  | (10.3-58.0) | (42.0-89.7) | (0.72-9.23) | (0.24-4.05) |
| **Current Smokeless Tobacco use** |  |  |  |  |  |
| Non-current | 192 | 42.9 | 57.1 | 1 | 1 |
|  |  | (32.7-53.8) | (46.2-67.3) |  |  |
| Current | 38 | 68.5 | 31.5 | 0.35 | 0.24 |
|  |  | (47.4-84.0) | (16.0-52.6) | (0.14-0.88) | (0.08-0.70) |
| **Hazardous drinking** |  |  |  |  |  |
| No | 218 | 48.6 | 51.4 | 1 | 1 |
|  |  | (38.7-58.7) | (41.3-61.3) |  |  |
| Yes | 12 | 21.1 | 78.9 | 3.54 | 1.44 |
|  |  | (2.7-72.1) | (27.9-97.3) | (0.37-33.84) | (0.19-10.80) |
| **Dietary salt related practices** |  |  |  |  |  |
| At least one* measure taken on regularly | 145 | 48.4 | 51.6 | 1 | 1 |
|  |  | (36.8-60.1) | (39.9-63.2) |  |  |
| None | 85 | 45.1 | 54.9 | 1.14 | 1.04 |
|  |  | (30.9-60.1) | (39.9-69.1) | (0.58-2.24) | (0.41-2.66) |
| **Co-morbidities** |  |  |  |  |  |
| No other self-reported chronic disease | 155 | 47.1 | 52.9 | 1 | 1 |
|  |  | (36.2-58.2) | (41.8-63.8) |  |  |
| Yes | 75 | 47.3 | 52.7 | 0.99 | 2.11 |
|  |  | (32.7-62.4) | (37.6-67.3) | (0.53-1.85) | (0.82-5.39) |
| **Adherence***** |  |  |  |  |  |
| ≥11 days | 136 | 55.3 | 44.7 | 1 | 1 |
|  |  | (43.6-66.4) | (33.6-56.4) |  |  |
| 6 to 10 days | 55 | 36.6 | 63.4 | 2.14 | 1.97 |
|  |  | (18.6-59.3) | (40.7-81.4) | (0.81-5.67) | (0.70-5.55) |
| ≤5 days | 39 | 33.7 | 66.3 | 2.43 | 3.52 |
|  |  | (17.4-55.2) | (44.8-82.6) | (0.90-6.58) | (1.04-11.91) |
| **Physicians consulted for Hypertension** |  |  |  |  |  |
| Govt. health facility | 51 | 43.5 | 56.5 | 1 | 1 |
|  |  | (26.9-61.7) | (38.3-73.1) |  |  |
| PVT/NGO health facility | 158 | 46.9 | 53.1 | 0.87 | 0.67 |
|  |  | (33.8-60.3) | (39.7-66.2) | (0.34-2.20) | (0.19-2.31) |
| No | 21 | 58.4 | 41.6 | 0.55 | 0.13 |
|  |  | (33.9-79.4) | (20.6-66.1) | (0.16-1.91) | (0.03-0.55) |
| **Source of medicines** |  |  |  |  |  |
| Govt. facility | 42 | 38.8 | 61.2 | 1 | 1 |
|  |  | (23.5-56.6) | (43.4-76.5) |  |  |
| Chemist/ Private/ NGO Dispensary | 180 | 48.1 | 51.9 | 0.68 | 0.96 |
|  |  | (36.6-59.9) | (40.1-63.4) | (0.30-1.54) | (0.22-4.09) |
| *Measures to control salt intake: Limit consumption of high salt containing food/Look at the salt or sodium content on food labels/use of low salt or sodium alternatives/Use spices other than salt/avoid foods prepared outside home/other measures. | | | | | |
| **Diabetes/ hypercholesterolemia / CVD | | | | | |
| ***adherence to treatment in last 2 weeks | | | | | |
